# Estimating Attractor Reachability in Asynchronous Logical Models

**DOI:** 10.3389/fphys.2018.01161

**Published:** 2018-09-07

**Authors:** Nuno D. Mendes, Rui Henriques, Elisabeth Remy, Jorge Carneiro, Pedro T. Monteiro, Claudine Chaouiya

**Affiliations:** ^1^Instituto Gulbenkian de Ciência, Oeiras, Portugal; ^2^Department of Computer Science and Engineering, Instituto Superior Técnico, Universidade de Lisboa, Lisbon, Portugal; ^3^Instituto de Engenharia de Sistemas e Computadores Investigação e Desenvolvimento, Lisbon, Portugal; ^4^Aix Marseille University, CNRS, Centrale Marseille, I2M UMR 7373, Marseille, France

**Keywords:** regulatory network, logical modeling, discrete asynchronous dynamics, attractors, reachability

## Abstract

Logical models are well-suited to capture salient dynamical properties of regulatory networks. For networks controlling cell fate decisions, cell fates are associated with model attractors (stable states or cyclic attractors) whose identification and reachability properties are particularly relevant. While synchronous updates assume unlikely instantaneous or identical rates associated with component changes, the consideration of asynchronous updates is more realistic but, for large models, may hinder the analysis of the resulting non-deterministic concurrent dynamics. This complexity hampers the study of asymptotical behaviors, and most existing approaches suffer from efficiency bottlenecks, being generally unable to handle cyclical attractors and quantify attractor reachability. Here, we propose two algorithms providing probability estimates of attractor reachability in asynchronous dynamics. The first algorithm, named Firefront, exhaustively explores the state space from an initial state, and provides quasi-exact evaluations of the reachability probabilities of model attractors. The algorithm progresses in breadth, propagating the probabilities of each encountered state to its successors. Second, Avatar is an adapted Monte Carlo approach, better suited for models with large and intertwined transient and terminal cycles. Avatar iteratively explores the state space by randomly selecting trajectories and by using these random walks to estimate the likelihood of reaching an attractor. Unlike Monte Carlo simulations, Avatar is equipped to avoid getting trapped in transient cycles and to identify cyclic attractors. Firefront and Avatar are validated and compared to related methods, using as test cases logical models of synthetic and biological networks. Both algorithms are implemented as new functionalities of GINsim 3.0, a well-established software tool for logical modeling, providing executable GUI, Java API, and scripting facilities.

## 1. Introduction

Logical modeling has been widely used to study gene regulatory and signalling networks (see e.g., Glass and Siegelmann, 2010; Saadatpour and Albert, 2012; Abou-Jaoudé et al., 2016). Briefly, in a logical model, the evolution of the discretised level of each component depends on the current values of its regulators whose influences are dictated by logical functions. Here, we rely on the generalized framework initially introduced by Thomas and d'Ari (1990) and implemented in our software tool GINsim (Chaouiya et al., 2012; Naldi et al., 2018). Because precise knowledge of the durations of underlying mechanisms is often lacking, one assumes that, when multiple components are called to change their levels, all update orders have to be considered. This corresponds to the asynchronous updating scheme (Thomas and d'Ari, 1990; Thomas, 1991). The dynamics of these models are classically represented by State Transition Graphs (STGs) where nodes embody the model states and edges represent the state transitions; each path in this graph accounts for a potential trajectory of the system. In contrast, synchronous updates, which amount to consider equal or negligible delays associated to component changes, define deterministic dynamics, easier to analyse but less realistic.

Model attractors (stable states or cyclic attractors) represent long term, stable equilibria. Cyclic attractors denote stable oscillations as observed in cell cycle or circadian rythms (see e.g., Fauré et al., 2006; Fauré and Thieffry, 2009; Chaves and Preto, 2013), whereas stable states are associated with cell lineages or other cellular responses to external cues or perturbations (see e.g., Sánchez et al., 2008; Calzone et al., 2010; Naldi et al., 2010; Collombet et al., 2017). Modeling molecular networks involved in cancer has been focusing on attractors and their reachability properties (see e.g., Huang et al., 2009; Flobak et al., 2015; Remy et al., 2015; Cho et al., 2016). Indeed, attractor likelihood may provide relevant predictions as attractors reflect cellular responses (e.g., healthy or not). For instance, to uncover patterns of genetic alterations in bladder tumors, Remy et al. (2015) considered an asynchronous logical model and checked how model perturbations modify the probabilities of reaching attractors related to proliferative phenotypes.

Not surprisingly, the number of states of logical models grows exponentially with the number of regulatory components. Moreover, due to the asynchronous updating scheme, the dynamics are non-deterministic; they possibly encompass alternative trajectories toward a given state as well as transient cycles. All this turns the identification and reachability analysis of model attractors into a difficult challenge. In this context, methods have been developed to find stable states—also referred as point attractors—and complex, oscillatory attractors (or, at least to circumscribe their location) (Naldi et al., 2007; Garg et al., 2008; Zañudo and Albert, 2013; Klarner et al., 2015). Here, we primarily aim at efficiently determining attractors reachable from specific initial condition(s) as well as estimating the reachability probability of each of those attractors in asynchronous dynamics.

An STG can be readily interpreted as the transition matrix of a finite Markov Chain. Generally, STGs encompass distinct attractors (or recurrent classes) and thus define absorbing chains (Grinstead et al., 1997). However, most existing results relate to recurrent (or irreducible) chains (Prum, 2012). Moreover, we aim at avoiding the construction of the whole dynamics (or the associated transition matrix); we thus rely on the logical rules as implicit descriptions of state transitions. Finally, we have here a specific interest on reachability properties.

Following a background section, we present two approaches to assess reachable attractors. First, the Firefront algorithm is a quasi-exact method that starts from an initial state and simultaneously follows all (concurrent) trajectories while propagating state probabilities. This algorithm follows a principle similar to those employed for infinite Markov chains (Munsky and Khammash, 2006; Henzinger et al., 2009). To enable state space sampling and tackle models with large transient cyclic behaviors, we developed Avatar, which is a Monte Carlo approach adapted to cope with strongly connected components. Both methods have been implemented as new functionalities of the software tool GINsim (Naldi et al., 2018). They are applied to a range of models, illustrating their respective performances and specificities.

## 2. Methods

In this section, we first briefly introduce the basics on Logical Regulatory Graphs (LRGs), their state transition graphs (STGs), attractors as well as absorbing Markov chains. We then present the algorithm Firefront. The rest of the section focuses on Avatar, an adaptation of the classical Monte Carlo simulation to cope with cyclical behaviors. It is worth noting that for small enough models it is possible to explicitly construct the STGs and identify reachable attractors, but it is not straightforward to evaluate their reachability probabilities.

### 2.1. Background

#### 2.1.1. Basics on logical models and their dynamics

*Definition 1*. A Logical Regulatory Graph (LRG) is a pair (*G, K*), where:

- *G* = {_*g*_*i*_}*i* = 0, …*n*_ is the set of regulatory components. Each *g*_*i*_ ∈ *G* is associated to a variable *v*^*i*^ denoting its level, which takes values in *D*_*i*_ = {0, …*M*_*i*_} ⊊ ℕ; $v={\left(v^{i}\right)}_{i=0,\ldots n}$ is a state of the system, and *S* = ∏_*i* = 0, …*n*_*D*_*i*_ denotes the state space.
- (_*K*_*i*_)*i* = 0, …*n*_ denotes the *logical regulatory functions* (or logical rules); *K*_*i*_:*S* → *D*_*i*_ is the function that specifies the evolution of *g*_*i*_; ∀*v* ∈ *S, K*_*i*_(*v*) is the target value of *g*_*i*_ that depends on the state *v*.

The asynchronous dynamics of an LRG is represented by a graph as follows.

*Definition 2*. Given a logical regulatory graph (*G, K*), its asynchronous State Transition Graph (STG) is denoted (*S, T*), where:

- *S* is the state space,
- *T* = {(*v, v*′) ∈ *S*^2^∣*v*′ ∈ Succ(*v*)}, where for each state *v*, Succ(*v*):*S* → 2^*S*^ is the set of successor states *w*, satisfying the asynchronous property (one component is updated at a time):
  ∃gi∈G with{Ki(v)=vi and wi=vi+Ki(v)−vi|Ki(v)−vi|,∀gj∈G∖{gi}, wj=vj.

Note that, from the STG defined above, one can consider the sub-graph reachable from a specific initial state *v*_0_ or from a set of states {_*v*_*i*_}*i* ∈ {0, …*m*}_ ⊆ *S*.

We further introduce some notation and classical notions.

Given an STG (*S, T*), we write *v* → *v*′ if and only if there exists a path between the states *v* and *v*′. In other words, there is a sequence of states of *S* such as: $v_{0}=v,v_{1},\ldots v_{k-1},v_{k}=v^{\prime }$, and for all *j* ∈ {1, …*k*}, (*v*_*j*−1_, *v*_*j*_) ∈ *T*. Furthermore, we denote $v\overset{k}{\to }v^{\prime }$ such a path of length *k*.

A Strongly Connected Component (SCC) is a maximal set of states *A* ⊆ *S* such that ∀*v, v*′ ∈ *A* with *v*≠*v*′, *v* → *v*′. This is to say, there is a path between any two states in *A*, and this property cannot be preserved adding any other state to *A*.

Attractors of an LRG are defined as the *terminal* SCCs of its STG (i.e., there is no transitions leaving the SCC). If a terminal SCC is a single state we call it a *stable state*, otherwise it is a *complex attractor*.

#### 2.1.2. Markov chains and absorption

The incidence matrix of an STG (*S, T*) naturally translates into an |*S*| × |*S*|-transition matrix Π, which is a stochastic matrix (for all *v* ∈ *S*, $\underset{u\in S}{\sum }\Pi \left(v,u\right)=1$):

$$\begin{array}{l} \forall v,v^{\prime }\in S\Pi \left(v,v^{\prime }\right)>0\Leftrightarrow \left(v,v^{\prime }\right)\in T,\\ \forall v\in S\Pi \left(v,v\right)=1\Leftrightarrow Succ\left(v\right)=\emptyset ,\\ \Pi \left(v,v\right)=0\text{otherwise}. \end{array}$$

We assume that probabilities of concurrent transitions are uniformly distributed: ∀*v* ∈ *S*, ∀*v*′ ∈ *Succ*(*v*), Π(*v, v*′) = 1/|*Succ*(*v*)|. Extension to other distributions would be rather straightforward.

A Markov chain (μ_0_, Π) is defined by the finite set *S*, the transition matrix Π, and the initial law μ_0_ (that depends on the selection – or not – of an initial condition). We want to define the chain stopped when it reaches an attractor. For that, we consider the quotient graph of (*S, T*) with respect to the equivalence relation: *u* ~ *v* ⇔ *u* → *v*and*v* → *u*. In this quotient graph, each node gathers a set of states and corresponds to a class of the Markov chain. The absorbing nodes of the quotient graph (i.e., nodes with no output arcs) form the absorbing classes of the chain (μ_0_, Π), all the other classes being transient. Note that the number of absorbing classes is the number of attractors of the corresponding STG. Let θ be this number and *a*_1_, …*a*_θ_ the absorbing classes.

Now, let us stop the chain (μ_0_, Π) when it reaches an absorbing class: we thus define the Markov chain *X* on the set $\tilde{S}=T\cup A$, where T⊂S is the set of all the transient states, and $A=\left\{\left\{a_{i}\right\},i=1,\ldots \theta \right\}$ (each element *a*_*i*_ being an absorbing class). The transition matrix π of *X* is:

$$\begin{array}{l} \pi \left(u,a_{i}\right)=\underset{v\in a_{i}}{\sum }\Pi \left(u,v\right)\;\forall u\in T,\forall a_{i}\in A,\\ \pi \left(a_{i},u\right)=0\;\forall u\in T,\forall a_{i}\in A,\\ \pi \left(a_{i},a_{i}\right)=1\;\forall a_{i}\in A,\\ \pi \left(a_{i},a_{j}\right)=0\;\forall a_{i}\in A,\forall a_{j}\in A,i\neq j,\\ \pi \left(u,v\right)=\Pi \left(u,v\right)\;\forall u,v\in T. \end{array}$$

Reordering the states by considering first the transient ones, (i.e., those belonging to T) and then the absorbing classes (i.e., the elements of A), the transition matrix π is under its canonical form:

$$\begin{array}{l} \pi =\left(\begin{array}{cc} Q & L\\ 0 & I \end{array}\right), \end{array}$$

where *Q*(*u, v*) = π(*u, v*) for u,v∈T, *L*(*u, a*) = π(*u, a*) for u∈T and a∈A, 0 is the null matrix (no transition from an absorbing class to a transient state), and *I* the identity matrix. One can easily verify that:

$$\begin{array}{l} \pi^{k}=\left(\begin{array}{cc} Q^{k} & \left(\sum_{j=0}^{k-1}Q^{j}\right)L\\ 0 & I \end{array}\right), \end{array}$$

π^*k*^(*u, v*) denotes the probability that, started in state *u*, the chain is in state *v* after *k* steps: $\pi^{k}\left(u,v\right)={\mathbb{P}}_{u}\left(X_{k}=v\right)\overset{\Delta }{=}\mathbb{P}\left(X_{k}=v|X_{0}=u\right)$. Proofs of the next, well-known results can be found in [e.g., (Grinstead et al., 1997), chap. 11].

- *Q*^*k*^ tends to 0 when *k* tends to infinity, and
  (1) $$\begin{array}{r} \underset{n\to +\infty }{\lim}\overset{n}{\underset{k=0}{\sum }}Q^{k}={\left(I-Q\right)}^{-1}. \end{array}$$
- The hitting time of A is almost-surely finite.
- From any u∈T, the probability of *X* being absorbed in a∈A is ${\mathbb{P}}_{u}\left(X_{\infty }=a\right)={\left(Id-Q\right)}^{-1}L\left(u,a\right).$

By an abuse of terminology, we will refer to ℙ_*u*_(*X*_∞_ = *a*) as the probability to reach the attractor *a* from the initial state *u*.

## 2.2. Firefront

Firefront is our first method to identify attractors and assess their reachability probabilities. Although simple, it is effective for restricted types of dynamics as demonstrated in section 4. Briefly, the algorithm progresses in breadth from an initial state *v*_0_, which is first assigned probability 1. It distributes and propagates the probability of each visited state to its successors, according to the transition matrix Π.

At any step *k*, the set of states being expanded and carrying a fraction of the original probability is called *firefront* as it corresponds to the front line of the breadth-first exploration of the STG: $F_{k}=\left\{v\in S,\exists v_{0}\overset{k}{\to }v\right\}$. Basically this procedure, called expansion, calculates at each iteration *k* and for each state *v* the probability of the Markov chain *X* to be in *v* after *k* steps from state *v*_0_: ${\mathbb{P}}_{v_{0}}\left(X_{k}=v\right)=\pi^{k}\left(v_{0},v\right)$. Clearly, by the definition of the set *F*_*k*_, ℙ_*v*_0__(*X*_*k*_ ∈ *F*_*k*_) = 1; the firefront will ultimately contain only states that are stable states or members of complex attractors. In what follows, we will simply denote the firefront set *F*, omitting the index *k*. Actually, attractors are not kept in *F*, they are instead stored in another set *A* (see below), hence *F* becomes ultimately empty.

**Figure 1:**
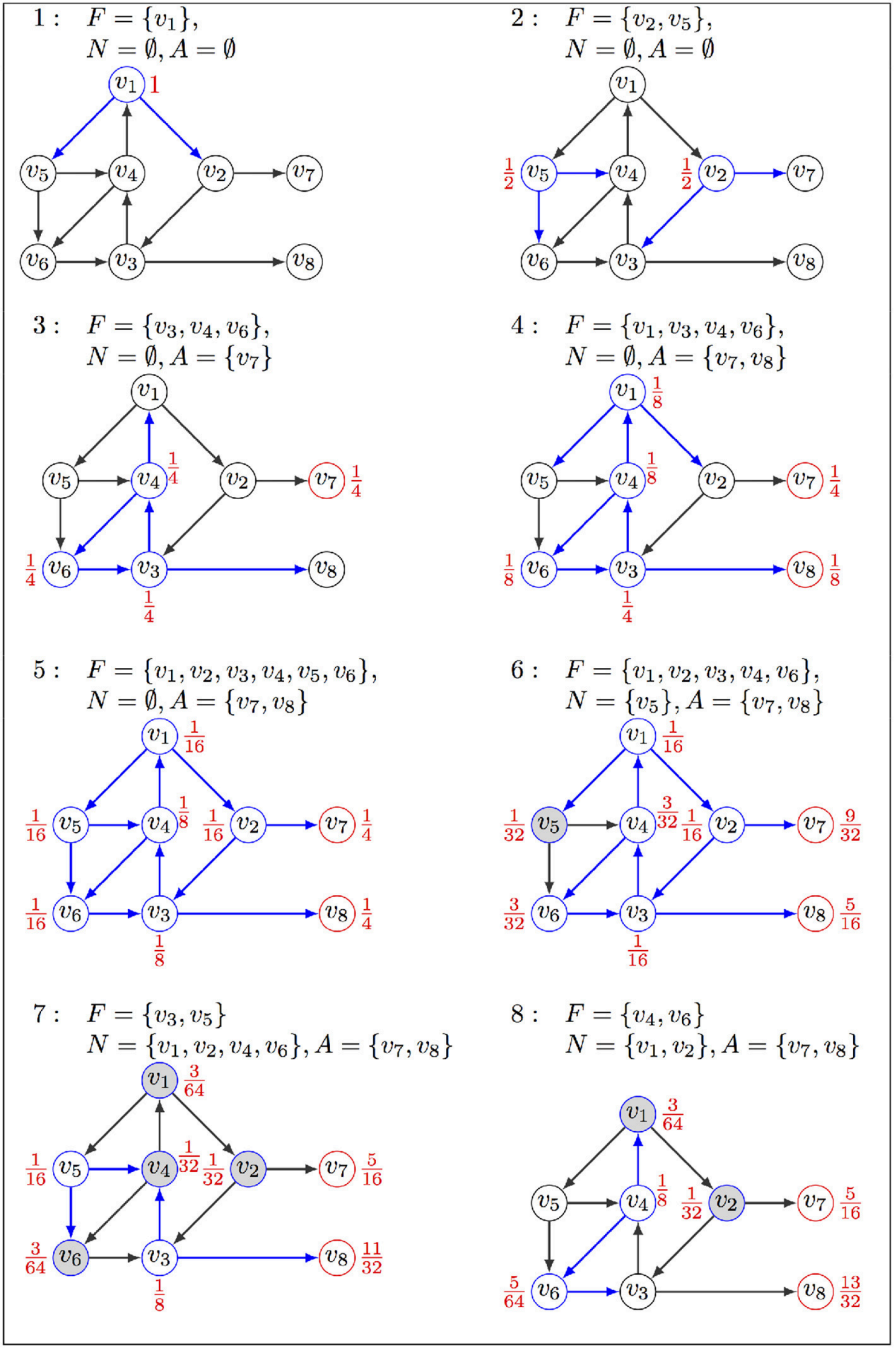
Illustration of firefront operation, with $\alpha =\frac{1}{16}$: (1) The exploration starts from initial state *v*_1_ in *F* associated with probability 1, sets *A* and *N* are empty; (2) successors replace *v*_1_ in *F*, associated with their probabilities; (3–4) states in *F* are replaced by their successors, but the stable state *v*_7_ goes in *A*; (4) *v*_3_, *v*_4_, *v*_6_ stay in *F* with updated probabilities; (5) probability of *v*_8_ in *A* increases as it is visited again; (6) *v*_5_ goes to *N* as its probability is lower than α; (7) *v*_5_ is removed from *N* and put back in *F* as its probability increased when visited again from *v*_1_. Transitions explored in the current iteration are in blue, their sources being labeled with their probabilities. Red nodes are in *A*, and gray nodes are in *N*. The exploration will halt when *F* is empty or the maximum number of iterations is reached.

In practice, to tackle efficiency bottlenecks avoiding the exploration of unlikely trajectories, we introduce a set of *neglected states N*. Furthermore, to ensure that the algorithm terminates whenever the reachable attractors are all stable states, we consider the set of *attractors A*. In the course of the exploration the firefront *F* is reduced as explained below:

- if the probability associated with a state *v* ∈ *F* drops below a certain value α, then *v* is moved from *F* to *N* (set of neglected states). As a consequence, the immediate successors of *v* will not be explored at this time. If a state *v* ∈ *N* is visited again as being the successor of a state in *F*, its probability is properly updated (we will say that it accumulates more probability), and if this probability exceeds α, then *v* is moved from *N* back to *F* (see Figure 1, step 7);
- if a state in *F* has no successors, it is moved to *A* (set of stable states); if it is already in *A*, its probability increases according to this new trajectory.

At each step, the sum of the probabilities of the states in *F*, *N*, and *A* is 1.

Unlike forest fires, which do not revisit burnt areas, the algorithm will, in general, revisit the same state in the presence of a cycle. This invalidates our colorful metaphor unless imagining uncannily rapid forest regeneration. The presence of cycles thus poses some difficulties because the algorithm would never terminate. To address this issue, Firefront detects periodicities of the ensemble of states entering and exiting *F* (i.e., states with a sustained oscillating probability); three sequential occurrences of exactly the same set *F* are assumed to be sufficient evidence that the simulation is locked within a complex attractor. In this situation, all the states found in *F* between the second and third occurrences are used to compose the complex attractor. To do so efficiently, Firefront uses a reversible hash-function. This heuristic thus enables the identification of complex attractors from oscillating behaviors throughout expansions. Nevertheless, since Firefront progression can still become locked in large and complex cycles for a lengthy number of expansions, the user may specify a maximum depth (number of expansions) to guarantee its termination in useful time.

When available, the algorithm can be provided with a description of the complex attractors, equipping Firefront with a function called *oracle* that indicates whether a state belongs to a listed complex attractor. In this case, Firefront halts the exploration whenever it reaches a state recognized by the oracle, and treats all members of the corresponding attractor as a single element of *A* collectively accumulating incoming probabilities.

Firefront terminates when: 1) the total probability in *F* drops to zero or below some predefined threshold β, or 2) the predefined maximum depth is reached. Given the initial state *v*_0_, the probability associated to each attractor *a* ∈ *A* is a lower bound of ℙ_*v*_0__(*X*_∞_ = *a*). An upper bound is obtained by adding to this value β and the sum of probabilities accumulated in *N*. An outline of Firefront is presented in Algorithm 1, and Figure 1 provides an illustration on a toy example.

**Algorithm 1:**
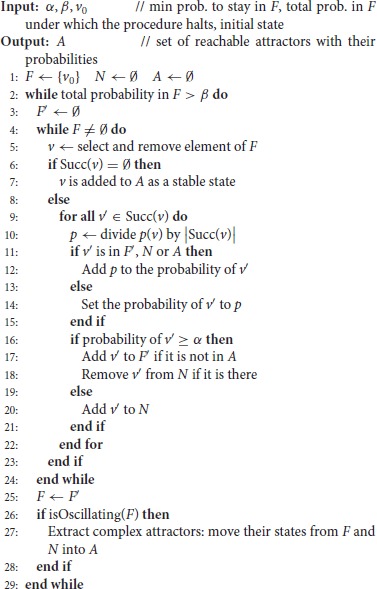
Firefront

## 2.3. Avatar

Avatar is proposed as an alternate algorithm to identify model attractors and quantify their reachability, considering specific initial state(s) or the whole state space. Avatar is an adaptation of the classical Monte Carlo simulations that aims at efficiently coping with (transient and terminal) SCCs.

### 2.3.1. The algorithm

When exhaustive enumeration is not feasible, Monte Carlo simulation is classically used to estimate the likelihood of an outcome. Concerning attractor reachability in logical models, this means following random paths along the asynchronous dynamics (the STG). Each simulation halts when either a stable state (with no successor) or the maximal depth are reached. Performing a large number of simulations allows estimating reachability probabilities of stable states. The simulation does not record past states, and thus memory requirements are minimal. However, a major drawback is that cycles are not detected. Consequently, without restricting the number of steps, the simulation does not terminate when a trajectory enters a terminal SCC. Moreover, in the presence of a transient cycle, it may re-visit the same states an unbounded number of times before exiting. That is why we propose an appropriate modification of this approach.

**Figure 2:**
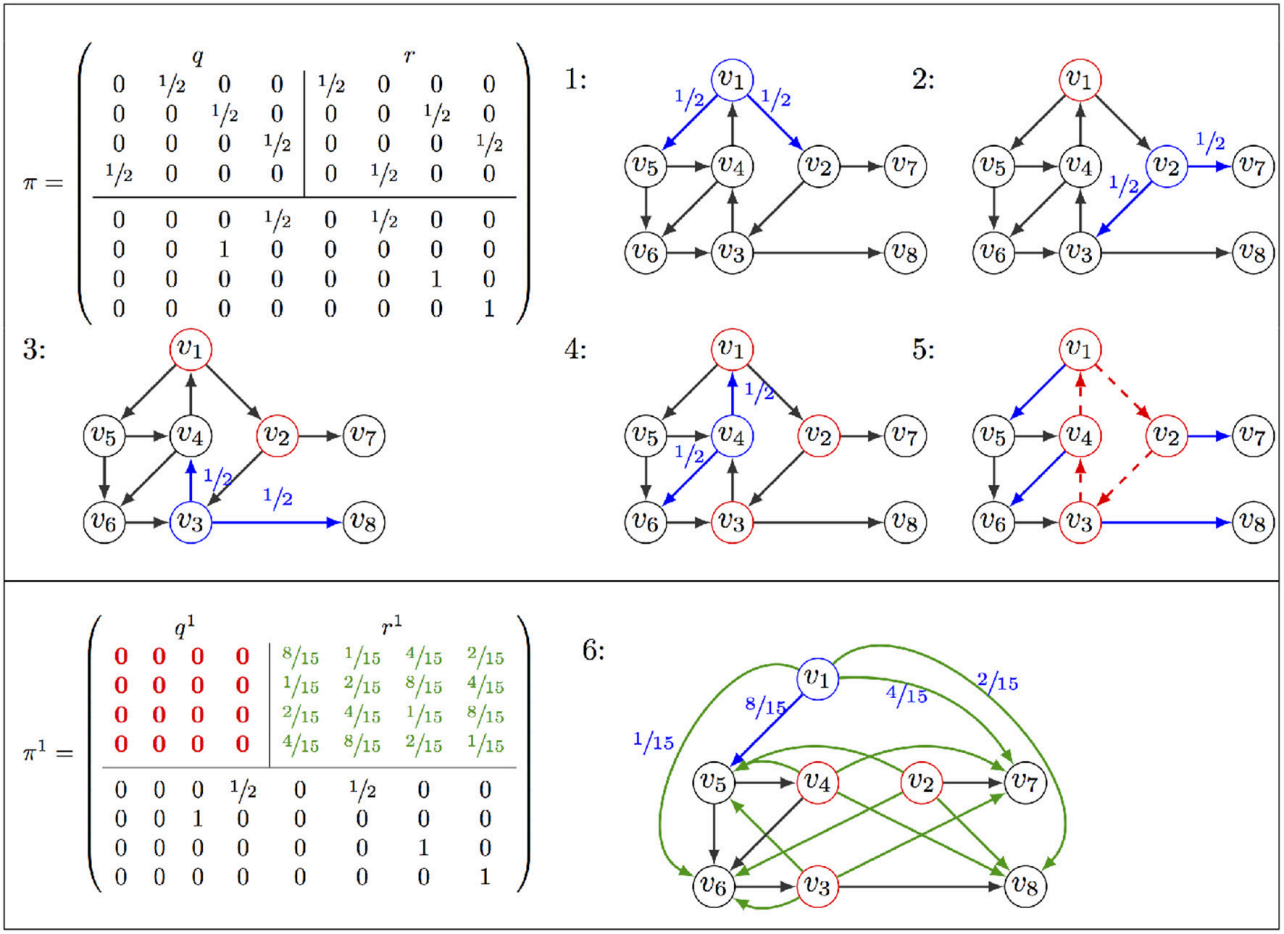
Illustration of avatar operation: The transition matrix π is partitioned into the sub-matrices *q* for transitions between states *v*_1_, …*v*_4_ of the cycle to be discovered **(Top Left)**, and *r* for transitions leaving the cycle **(Top Right)**. Exploration starts at *v*_1_ (denoted in blue as well as its leaving transitions with their probabilities), *v*_2_ is selected for the second iteration, and *v*_1_ is indicated as being already visited in red. Exploration proceeds until revisiting *v*_1_ at the 5^*th*^ step. Having identified a cycle, the rewiring procedure is launched, removing transitions of the cycle (dotted red) and adding transitions toward exits (green). Probabilities are computed, resulting in a new matrix π^1^, with $q_{ij}^{1}=0$ and $r_{ij}^{1}={\left({\left(Id-q\right)}^{-1}r\right)}_{ij},i=1,\ldots 4$. From *v*_1_, an exit of the cycle is chosen according to these probabilities (step 6).

Avatar is outlined in Algorithm 2 (further description of Avatar and its ancillary procedures is provided in the Supplementary Material S1). It avoids repeatedly visiting states by detecting that a previously visited state is reached, indicating the presence of a cycle in the dynamics. Having detected a cycle, the algorithm modifies the STG in order to dismantle the cycle, linking its states to its exiting states (i.e., targets of transitions leaving the cycle). It is important, however, to associate these new transitions with appropriate probabilities; the probability of a transition from any cycle state to a given exit must match the corresponding asymptotic probability, considering the infinitely many possible trajectories. The STG is thus rewired so as to replace all the transitions between the cycle states by transitions from each cycle state toward each cycle exit (see Figure 2). Each rewiring creates a new so-called *incarnation* of the dynamics. Such an incarnation—Sanskrit name of our algorithm—is a graph with the same states as the original STG, but with different transition probabilities. This rewiring relies on theoretical foundations that are presented in section 2.3.2. Upon rewiring, the simulation proceeds from the current state.

**Algorithm 2:**
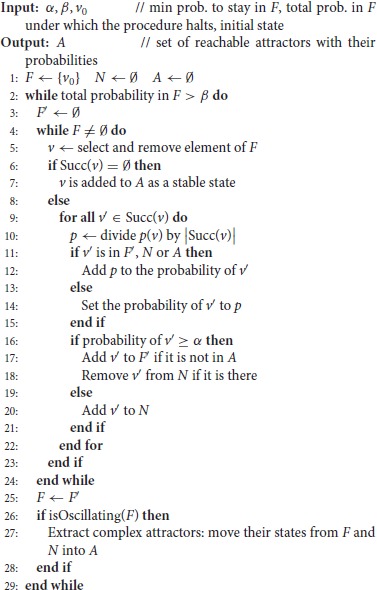
Avatar (single simulation)

Because it is generally more efficient to rewire a large transient than to iteratively rewire portions of it, upon encountering a cycle, Avatar performs an extension step controlled by a parameter τ that is a modified Tarjan's algorithm for SCC identification (Tarjan, 1972)—trajectories exploration is performed up to a depth of τ away from states of the original cycle. The subsequent rewiring is then performed over the (potentially) extended cycle. In the course of a single simulation, the value of τ is doubled within each attempt to enlarge a cycle in order to speed up the identification of large transients.

When a newly visited state *v* has no successor, it is a stable state. But if *v* was part of a cycle in a previous incarnation, *v* belongs to a complex attractor, which is computed as the equivalence class containing all the cycles that included *v* in past incarnations.

As for Firefront, the algorithm can be complemented with the previous knowledge of the attractors (oracles). This obviously improves Avatar's performance. Moreover, Avatar not only evaluates the probability of the attractors being reached from an initial condition, it can also be used to assess the probability distribution of the attractors for the whole state space (i.e., considering all possible initial states). Avatar is also able to use the knowledge regarding the identified transient SCCs within one iteration to alleviate the cost of identifying and possibly rewiring large cycles in upcoming iterations, thus boosting the overall efficiency of the simulation. The knowledge regarding the sizes of the transient SCCs and average depths of the found attractors can provide valuable insights into the model dynamics.

### 2.3.2. Theoretical foundations of Avatar rewiring

The rewiring performed by Avatar to force the simulation exiting a cycle modifies the probabilities associated to transitions. This is properly done so as to ensure a correct evaluation of the reachability probabilities performing a (large) number of random walks over our Markov chain *X*. This procedure amounts to modify the chain. It is formalized below and illustrated in Figure 2.

Suppose that *X*_*t*_ = *c*_1_, and *X*_*t*+*k*_ = *c*_1_ for *t* and *k* two positive integers. The walk has thus traveled along the cycle *C* = (*c*_1_, *c*_2_, …*c*_*k*_) (with *c*_*i*_ ∈ *S* and (*c*_*i*_, *c*_*i*+1_) ∈ *T*, ∀*i* = 1, …*k*). Note that this cycle may contain “direct shortcuts”: (*c*_*i*_, *c*_*j*_) ∈ *T*, *j* ≠ *i*+1 (mod *k*). We denote by *B* the set of states directly reachable from *C*: *B* = {*v* ∈ *S*\*C*, (*c*_*i*_, *v*) ∈ *T, c*_*i*_ ∈ *C*}. Let *q* be the *k* × *k* sub-matrix of π, for states *c*_1_, …*c*_*k*_, and *r* the *k*×|*B*| sub-matrix of π, defining transitions from *C* to *B*. To force the walk leaving the cycle (rather than being trapped there for a long time), the transition matrix is modified as follows:

- remove the transitions between the states of *C*; the sub-matrix *q* is replaced by *q*^1^ = 0, the null matrix;
- add an arc from each state of *C* to each state of *B*; the sub-matrix *r* is replaced by $r^{1}\overset{\Delta }{=}\overset{\infty }{\underset{j=0}{\sum }}q^{j}r$. By Equation (1), section 2.1.2, $\forall c_{i}\in C,\forall v\in B,r^{1}\left(c_{i},v\right)=\left[{\left(Id-q\right)}^{-1}r\right]\left(c_{i},v\right).$

*Y* denotes this new chain. Property 1 asserts that, starting from any transient state *u*, *X*, and *Y* have the same asymptotical behaviors.

Property 1. ∀u∈T, ∀a∈A, ℙ_*u*_(*Y*_∞_ = *a*) = ℙ_*u*_(*X*_∞_ = *a*).

*Proof*. Transition matrices of *X* and *Y* are the same except around the states of the cycle *C*; they behave differently only when traveling along *C*: from *c*_*i*_, entry state of *C*, *X* runs along *C* for *l* steps (*l* ≥ 0), leaving *C* through a state *v* ∈ *B* with probability $q^{l}r\left(c_{i},v\right)$, whereas *Y* would go directly from *c*_*i*_ to *v*, with probability $r^{1}\left(c_{i},v\right)$. Hence, for all u∈T, a∈A and *j* ≥ 0, we have ℙ_*u*_(*Y*_*j*_ = *a*) ≥ ℙ_*u*_(*X*_*j*_ = *a*) and thus,

$$\begin{array}{lll} \overset{k}{\underset{j=1}{\sum }}{\mathbb{P}}_{u}\left(Y_{j}=a\right) & \geq & \overset{k}{\underset{j=1}{\sum }}{\mathbb{P}}_{u}\left(X_{j}=a\right),\\ {\mathbb{P}}_{u}\left(Y_{\infty }=a\right) & \geq & {\mathbb{P}}_{u}\left(X_{\infty }=a\right),\\ 1=\underset{a\in A}{\sum }{\mathbb{P}}_{u}\left(Y_{\infty }=a\right) & \geq & \underset{a\in A}{\sum }{\mathbb{P}}_{u}\left(X_{\infty }=a\right)=1. \end{array}$$

All the terms being positive, the Property is proved. Therefore, the rewiring does not asymptotically affect the output of the simulation.

         □

Despite the inherent simplicity and time efficiency of the rewiring step, its dependency on matrix inversions can lead to a memory bottleneck for very large cycles. As such, the current implementation of Avatar uses a ceiling size for a cycle to be rewired. When Avatar finds a cycle, it still attempts to extend it as far as possible. If the extended cycle has some exits, it needs to be rewired. However, if the extended cycle has more states than the specified ceiling, only a sub-cycle (with as much states as allowed) of the detected cycle is rewired. Furthermore, the user can also choose an approximate strategy for rewiring that still guarantees the selection of exit states when entering a cycle without the need to perform an exact estimation of their likelihood. This is done by assigning uniform probabilities from the states of a cycle to its exits. Although this strategy is not prone to memory bottlenecks, its approximate nature can lead to biases on the computed reachability probabilities.

## 3. Implementation

**Figure 3:**
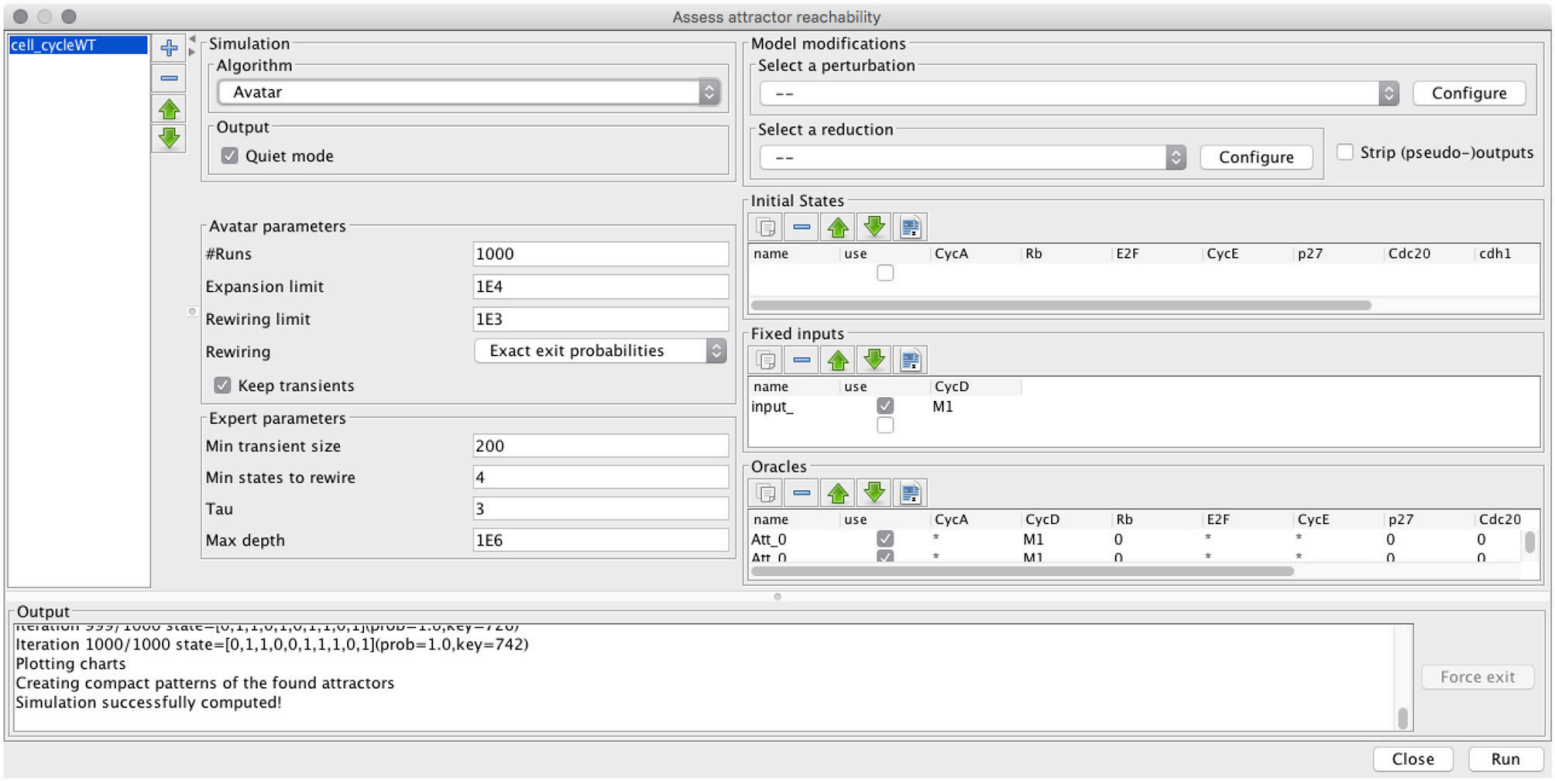
GUI for the assessment of attractor reachability within ginsim.

Both Firefront and Avatar are implemented in the context of GINsim, which supports the definition and analysis of logical models (Chaouiya et al., 2012; Naldi et al., 2018). Figure 3 provides a snapshot of the desktop GUI, showing the selection of the algorithm, specification of model modifications (perturbation or reduction), initial conditions, and algorithm parameters. MonteCarlo simulations are also available, as well as a modified version of Avatar with the approximate strategy described above. User documentation of Firefront and Avatar is provided in the Supplementary Material S2.

The implementations of Firefront and Avatar rely on adequate data structures—states are easily indexable through meaningful and compact hash keys, and sets of states are implemented as a map of states for highly efficient indexations, additions and removals. Our implementation of Firefront halts the STG exploration after a predefined number of expansions (10^3^ by default). Avatar implementation includes a heuristic optimization controlled by optional parameters whose default values were found to be appropriate for the tested models. This optimization considers tradeoffs between costly rewirings and simulations freely proceeding along cycles, as well as between memory cost of keeping state transitions after rewiring and not profiting from rewirings in previous simulations. Avatar further supports sampling over (portions of) the state space. In this case, iterations within a simulation start from states randomly selected over the unconstrained model components.

Both algorithms provide textual and visual displays of the results: attractors and their reachability probabilities, maximal size of encountered transient SCCs, and plots of the evolution of the set contents for Firefront and of the probability estimates for Avatar (see section 4).

## 4. Results

To validate the proposed algorithms, we considered a number of case studies including randomly generated, synthetic and published biological models. All are briefly described below. We analyzed how Firefront and Avatar perform on these case studies and compared, when possible, to outcomes produced by BoolNet (Müssel et al., 2010) and MonteCarlo simulations. BoolNet is an R package not only able to generate random Boolean models, but also to identify attractors and to perform Markov chain simulations. We further compared Avatar with MaBoSS, a C++ software implementing a Monte Carlo kinetic algorithm to produce time trajectories of Boolean models (Stoll et al., 2012), and with the probabilistic model checker Prism (Kwiatkowska et al., 2011, 2017). The experiments were run using an Intel(R) i7-7500U CPU @ 2.7GHz and 8GB of RAM.

### 4.1. Case studies description

Two sets of synthetic models were generated. First, we used BoolNet (Müssel et al., 2010) to define random models with 10 to 15 components, each with 2 regulators and logical rules randomly selected (uniform distribution)^1^. From the resulting set of random models, three models were selected for exhibiting multi-stability (Table 1). Additionally, we constructed a “synthetic” model exhibiting a large complex attractor and a few transient cycles. To further challenge our algorithms, we modified this last model, adding one component in such a way that the complex attractor turned into a transient cycle with very few transitions leaving toward a stable state (see synthetic models 1 and 2 in Table 1).

**Table 1:** Characteristics of the models used as case studies to challenge Firefront and Avatar: type of variables (Boolean vs. multi-valued), number of input components (these remain constant) and internal components, number and type of attractors with number of states in the case of complex attractors, size of the state space with the total number of model states.

**Model name**	**Boolean**	# Components		# Attractors		**# States**
	**(Y/N)**	**Input**	**Internal**	**Stable states**	**Complex attractors (*size*)**	
Random 1	Y	0	10	1	1 (4)	1 024
Random 2	Y	0	10	1	1 (4)	1 024
Random 3	Y	0	15	1	1 (4)	32 768
Synthetic 1	Y	0	15	1	1 (8192)	32 768
Synthetic 2	Y	0	16	2	0	65 536
Mammalian Cell Cycle	Y	1	9	1	1 (112)	1 024
Segment Polarity (*sp1*, 1-cell)	N	2	12	3	0	186 624
Segment Polarity (*sp2*, 2-cells)	N	0	24	3	0	≈9.7 × 10^8^
Segment Polarity (*sp4*, 4-cells)	N	0	48	15	0	≈9.4 × 10^17^
Bladder model	N	4	26	20	5 (16,16,32,512,184320)	≈8.5 × 10^9^

Our case studies also include published biological models. First, a Boolean model of the mammalian cell cycle control (Fauré et al., 2006), which has 10 components and exhibits one stable state (quiescent state) and one complex attractor (cell cycle progression). These attractors arise in (two) disconnected regions of the state space, controlled by the value of the sole input component (CycD, which stands for the presence of growth factors).

Second, Sanchez et al.'s multi-valued model of the segment polarity module—involved in early segmentation of the *Drosophila* embryo—defines an intra-cellular regulatory network. Instances of this network are connected through inter-cellular signaling (Sánchez et al., 2008). Here, we consider three cases: 1) the intra-cellular network (one cell), 2) the composition of two instances (i.e., two adjacent cells), and 3) the composition of four instances. Initial conditions are specified by the action of the pair-rule module (Wg-expressing cell for the single cell model) that operates earlier in development (see Sánchez et al., 2008 for details).

Third, we consider the interaction network of genes frequently altered in bladder cancer as proposed in Remy et al. (2015). This model includes 4 input components leading to different responses (EGFR, FGFR3 stimuli, Growth inhibitor, DNA damage), 23 internal components and 3 output components representing cellular responses or phenotypes (Proliferation, Apoptosis, Growth Arrest). Depending on the input values, the model displays multistability or not, with a combination of stable states and complex attractors. This case study further demonstrates the capacity of Avatar in assessing large complex attractors, quantifying attractor reachability, and revealing transient dynamics.

Finally, using a model of T helper cells differentiation (Naldi et al., 2010) and a model of cell fate decision in response to death receptor engagement (Calzone et al., 2010), we provide additional illustrations in the Supplementary Materials S4, S5.

Supplementary Material S6 provides an archive containing all the models in the GINsim format (zginml).

### 4.2. Firefront and Avatar in action

Results are summarized in Table 2. Generally, Firefront and Avatar show efficiency gains against alternatives and are further able to surpass the drawbacks of BoolNet (applicable to Boolean models only) and MonteCarlo (unable to identify transient and terminal cycles).

**Table 2:** Summary of the results for Firefront, Avatar, BoolNet, and MonteCarlo.

**Name (initial state)**		Firefront					Avatar				BoolNet			MonteCarlo			
	**# Reach**	**Time**	**Attract.**	**Prob. bounds**	**Residual**	**Expansions**	**Time**	**Attract.**	**Prob.**	**Largest transient**	**Time**	**Attract**.	**Prob.**	**Time**	**Attract.**	**Prob. (bounds)**	**Support (%simulations)**
*random1*(0000000000)	1024	1s	SS1 CA1	[0.674,0.678] [0.322,0.326]	4.2E-3	54	6 s	SS1 CA1	0.672 0.328	880	19 s	SS1 CA1	0.67 0.33	9 s	SS1	[0.91,1.00]	0.91
*random2*(0100011100)	88	1s	SS1 CA1	[0.25,0.25] [0.75,0.75]	1E-4	40	4 s	SS1 CA1	0.256 0.744	4	19 s	SS1 CA1	0.25 0.75	14 s	SS1	[0.77,1.00]	0.77
*random3*(100000000000000)	1408	1s	SS1 CA1	[0.21,0.21] [0.79,0.79]	3.4E-3	37	5 s	SS1 CA1	0.205 0.795	168	20 s	SS1 CA1	0.2 0.8	69 s	SS1	[0.08,1.00]	0.08
*synthetic1*(000001100110111)	28320	93s	SS1	[0.51,1.00]	0.49	10000	12 s	SS1 CA1	0.586 0.414	1024	8 days	SS1 CA1	0.6 0.4	72 s	SS1	[0.19,1.00]	0.19
*synthetic2*(0000001000000100)	16224	86s	SS1 SS2	[0.01,0.96] [0.05,1.00]	0.95	10000	421 s	SS1 SS2	0.92 0.08	8192	5 days	SS1 SS2	0.92 0.08	89 s	SS1	[0.006,1.00]	0.006
*mmc* quiescent & CycD=1	148	1s	CA1	[1.00,1.00]	7.8E-4	28	3s	CA1	1.00	4	195s	CA1	1.00	61s	–	–	0.0
*mmc* (sampling)	1024	NA due to sampling					2s	CA1 SS1	0.506 0.494	416	110s	CA1 SS1	0.5 0.5	65s	SS1	[0.51,1.00]	0.51
*sp1* (Wg-exp.)	330	0.2s	SS1 SS2	[0.84,0.84] [0.16,0.16]	8.4E-4	42	3s	SS1 SS2	0.837 0.162	76	NA (multivalued)			1s	SS1 SS2	0.810.19	1.0
*sp2* (pair rule)	3.2434E8	3s	SS1 SS2	[0.63,0.90] [0.10,0.37]	0.27	263	153s	SS1 SS2 SS3	0.8921 0.1078 1E-4	1844562	NA (multivalued)			1s	SS1 SS2 SS3	0.78 0.21 0.01	1.0
*sp4* (pair rule)	unknown	25s	SS1 SS2 SS3 SS4	[0.11,0.97] [0.02,0.88] [0.01,0.87] [0.00,0.86]	0.86	333	3074s	SS1 SS2 SS3 SS4 SS5 SS6–9	0.8645 0.0628 0.0571 0.0133 0.0016 < 1E-3	1841692	NA (multivalued)			5s	SS1 SS2 SS3 SS4 SS5 SS6–9	0.89 0.064 0.028 0.012 1E-3 < 1E-3	1.0

*Parameters: for* Firefront, *α = 10^−5^, β = 10^−5^ and maximum of expansions of 10^4^; for* Avatar *and* MonteCarlo, *number of runs of 10^4^ and maximum depth of 10^4^; for Avatar rewiring limit of 10^4^, expansion limit of 10^3^, growth factor τ = 3 and a minimum of 200 and 4 states to respectively save transients and apply rewiring. For* Firefront, *residual probabilities are the sums of those found in the firefront F and neglected N sets. Stable states are denoted SS and complex attractors CA*.

Considering **random models 1 to 3**, Firefront and Avatar are able to efficiently find the stable states and complex attractors of these models and to estimate their reachability probabilities. BoolNet is slower for these random models. MonteCarlo is not only less efficient but is also unable to detect the complex attractors. For instance, in random model 2, less than 8% of the simulations succeeded.

For **synthetic model 1**, Firefront takes over a minute to distribute the probability out of the large transient cycles. For **synthetic model 2**, Firefront could not distribute more than 5% of the probability out of the transient SCC (purposely constructed with 8 196 states and a dozen exits). The presence of multiple large transient SCCs causes Firefront to accumulate a large number of states in *F*, leading to some time overhead and difficulty to distribute the probabilities. States of transient SCCs are revisited until the probabilities of their incoming transitions drop below α, which can take long. As such, the computational performance of Firefront is greatly influenced by the structure of the STG (e.g., state outdegrees or sizes of transient SCCs). The Supplementary Material S3 provides illustrations of the structures of the dynamics. In contrast, Avatar is able to adequately identify and exit transient SCCs. For this reason, Avatar was able to escape the transient SCC planted in synthetic model 2 thanks to its rewiring procedure, and could identify and quantify the attractors for both synthetic models. BoolNet completed synthetic models 1 and 2, after 7 and 5 days, respectively, which highlights the need for the proposed methods to face efficiency bottlenecks for models with large and complex SCCs.

Starting in the region of the state space where the **mammalian cell cycle model** has a (unique) complex attractor (i.e., with the presence of CycD), Avatar, Firefront, and BoolNet could assess its reachability from the quiescent state; when sampling the state space, both Avatar and BoolNet could correctly quantify the reachability of the two attractors (Firefront was not applicable as it requires a starting initial state). Expectedly, MonteCarlo could not retrieve the complex attractor, being unable to exit it in all runs.

With regards to the **segment polarity model**, Firefront was efficient for all cases (single, two and four cells), although its ability to distribute all the probability decreases with the increase of model size. Since it did not reach the allowed maximum number of iterations, its stopping condition was that the total probability in *F* dropped below β, with all the residual probability in the neglected set, which in the end contained approximately 140, 52 000, and 210 000 states for the models of single, two and four cells, respectively. This would suggest that α was not small enough with respect to the number of concurrent trajectories toward the attractors (see Supplementary Material S4 for illustration). Although Avatar's performance is constrained by the need to assess the complex structure of the two and four cells' models (for instance the largest encountered transient SCC for *sp2* has over a million states), it is adequately able to find the attractors, even those with a low reachability probability. Given the fact that the attractors of these models are stable states, MonteCarlo was able to retrieve them, in particular those attractors reachable without the need to visit large transient SCCs.

**Figure 4:**
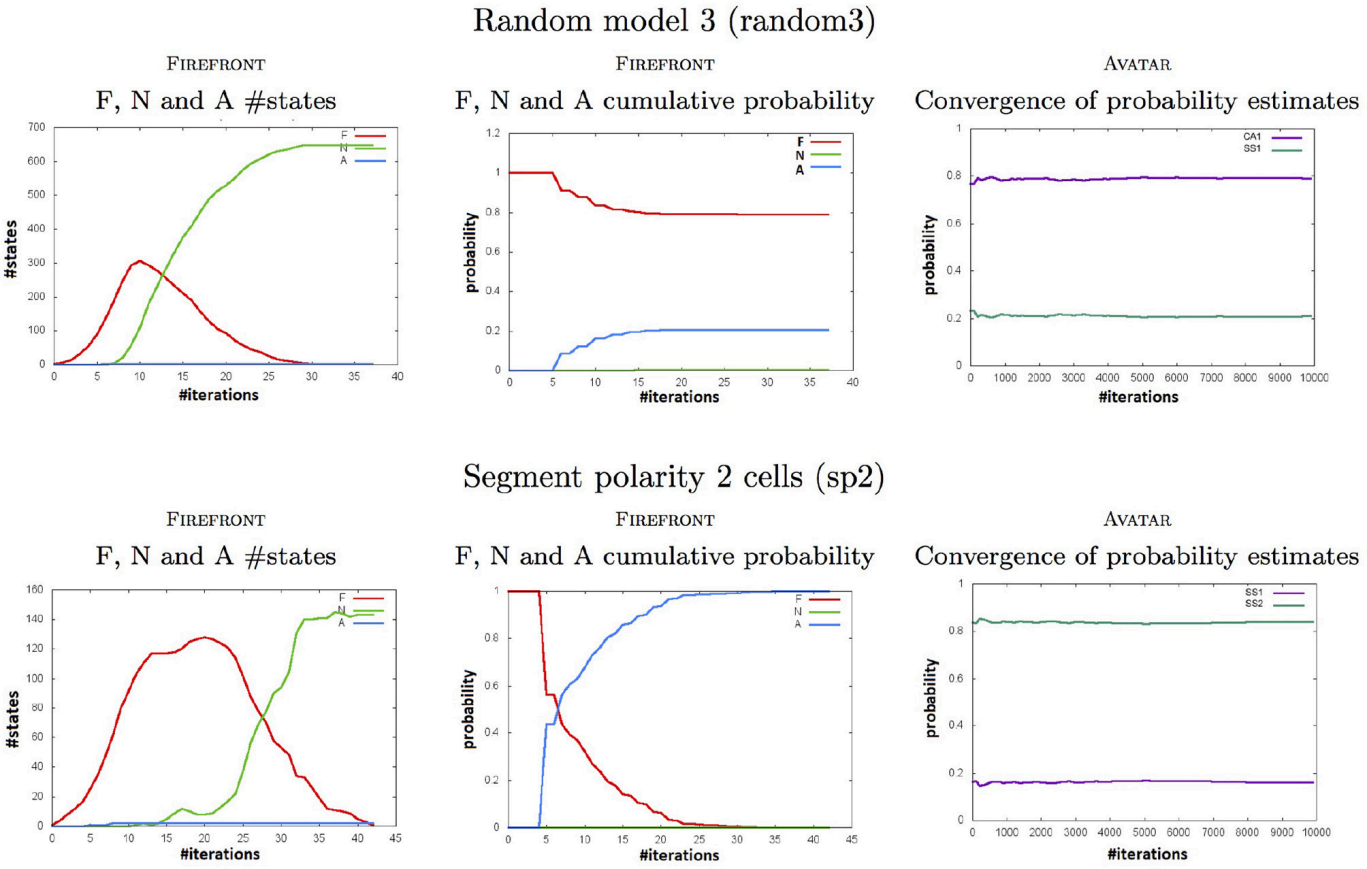
Plots computed by firefront and avatar throughout simulations for the *random3* (Top) and the *sp1* (Bottom) models (see Tables 1, 2). Left plots show the numbers of states to be expanded (in *F*), of neglected states (in *N*), and of attractors (in *A*). Middle plots show the cumulative probabilities of the 3 sets. Right plots show the convergence of the reachability probability of each attractor.

Figure 4 complements these results by showing, for two of our case studies: with Firefront, the evolution of the cardinals of the sets *F*, *N*, and *A* (and their corresponding probabilities), and with Avatar, the convergence of the estimated reachability probabilities of the attractors.

The application of Avatar over the **bladder tumourigenesis model**—with results illustrated in Table 3—enabled the quantification of attractor reachability over the whole state space, for 8 combinations of input values. Stable states were gathered in 3 classes, corresponding to the cell phenotypes Proliferation, Apoptosis and Growth Arrest, which are indicated by the values of the 3 output components of the model. The model displays several complex attractors. The reachability quantification of the attractors is relevant in the cases of multi-stability, i.e., when several attractors arise for the same input condition (compare with Table S2 in Remy et al., 2015). Avatar discloses structural properties of the model dynamics such as the sizes of encountered transient SCCs and mean depths of the attractors (not shown).

**Table 3:** Attractor analysis of the bladder tumourigenesis model performed with Avatar and MaBoSS.

**DNA damage**	**EGFR stimulus**	**FGFR3 stimulus**	**Growth inhibitor**	Avatar				MaBoSS		
				**Time**	**Attractors**	**Prob.**	**Largest SCC**	**Time**	**Attractors**	**Prob.**
0	0	0	0	162s	GA1	1.00	163528	15.76s	GA1	1.00
0	0	0	1	284s	GA2 GA3	0.882 0.118	239994	15.81s	GA2 GA3	0.8850.115
0	0	1	0	373s	Pr1	1.00	253440	13s	Pr1	1.00
0	0	1	1	258s	GA4 GA5 Pr2	0.770 0.095 0.135	135483	14.95s	GA4 GA5 Pr2	0.7220.1210.157
0	1	0	0	382s	Un1 (#184320)	1.00	184320	699s	—	—
0	1	0	1	421s	GA6 (#512)	1.00	242486	457.57s	—	—
0	1	1	0	212s	Pr1	1.00	151435	11.14s	Pr1	1.00
0	1	1	1	176s	GA4 GA5 Pr2	0.775 0.070 0.155	289593	11.2s	GA4 GA5 Pr2	0.7370.10.162

*The eight input configurations with DNA damage at 0 are considered. Attractors are named depending on the corresponding phenotype: Pr for Proliferation, GA for Growth Arrest, Ap for Apoptosis, Un for Undecided (output components are oscillating). Attractor sizes are indicated for complex attractors. Attractor probabilities are estimated over the whole state space*. Avatar *parameters: 10^3^ runs, up to 10^6^ states for expansion, up to 10^3^ states for rewiring, and 10^8^ maximum depth.* MaBoSS *parameters: time tick=1.0; max time=10^4^; sample count=10^4^; discrete time=1*.

**Figure 5:**
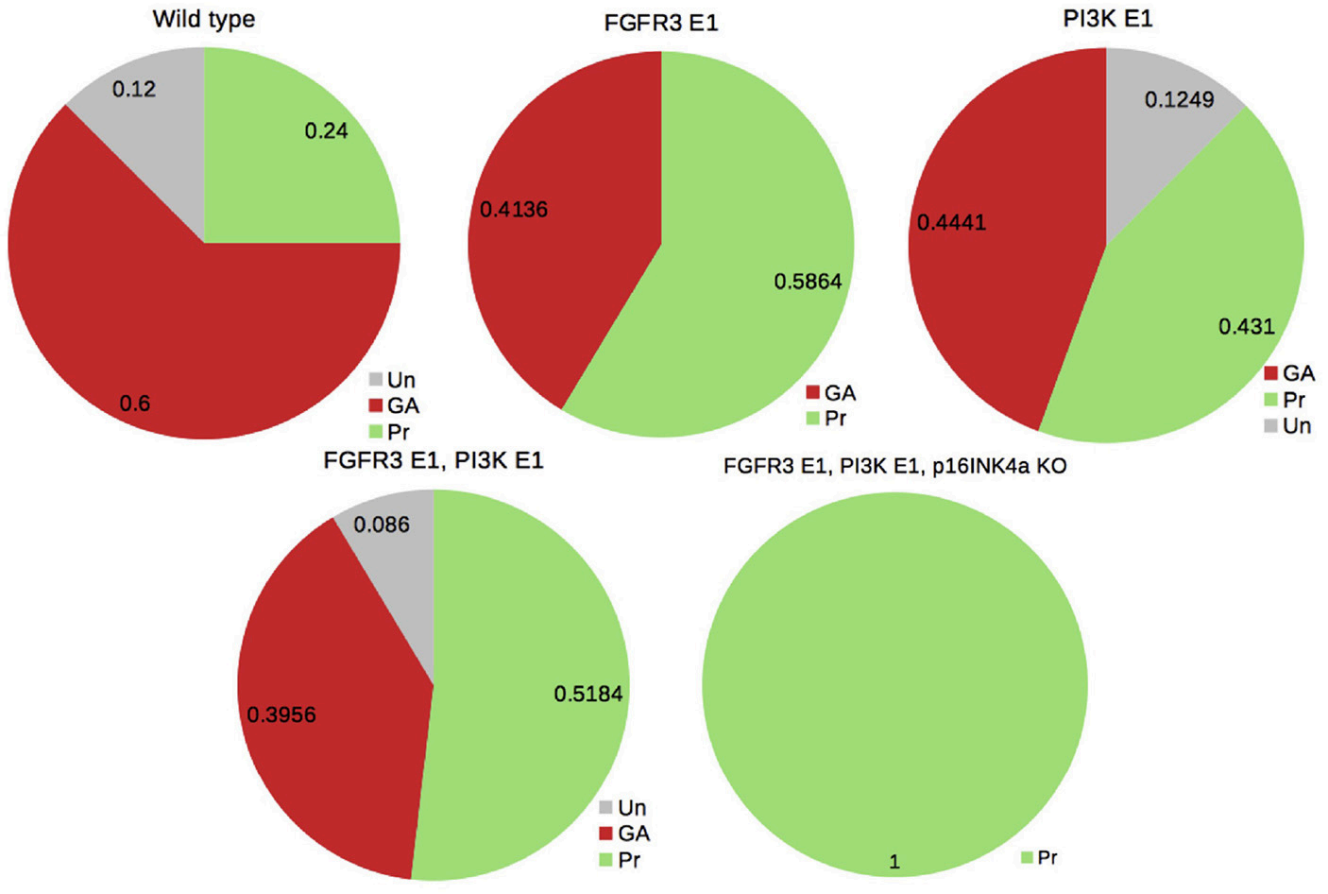
Probabilities of the phenotypes for the bladder tumourigenesis model in the wild type and mutant contexts: probabilities for the double mutant FGRF3 overexpression (FGFR3 E1) and PI3K overexpression (PI3K E1) suggest a slight advantage in mutating FGFR3 in a PI3K-mutated context (by increasing the probability of Proliferation); a third mutation of the tumor suppressor CDKN2A (coding for p16INKa) leads to the sole phenotype Proliferation (see Remy et al., 2015).

We also performed the analysis of model perturbations to illustrate the biological relevance of assessing attractor probabilities. To this end, we considered the case of activating mutations of fibroblast growth factor receptor 3 (FGFR3) and of the oncogene PI3K, one of the co-occurrent genetic perturbations observed in bladder tumors (see Remy et al., 2015). Figure 5 illustrates how probabilities of the attractors are modified under those perturbations. It supports the conclusions drawn in Remy et al. (2015): mutating FGFR3 in PIK3-mutated tumors seems to be advantageous (to increase the probability of Proliferation); a third mutation is required for uncontrolled proliferation (i.e., the loss of all the phenotypes but Proliferation).

For completeness, we also compared Avatar with MaBoSS and Prism. For this, we used GINsim export facilities of logical models to MaBoSS and Prism formats.

MaBoSS is a related command-line tool that generalizes Boolean models by defining stochastic rates associated with component updates (Stoll et al., 2012). MaBoSS primary goal is to compute temporal evolutions of state probability distributions and to estimate stationary distributions. To this end, it relies on the Gillespie algorithm. MaBoSS is thus well suited to get a quantitative view of temporal evolutions in the form of stochastic trajectories (see e.g., Abou-Jaoudé et al., 2016). When running MaBoSS on our case studies, it appeared that the tool was able to provide the reachability probabilities of the stable states of the random models 1 to 3. However, the presence of large transient SCCs or of complex attractors hinders the evaluation of such a measure for the synthetic models and for the cell cycle model. Table 3 includes the results obtained with MaBoSS for the analysis of the **bladder tumourigenesis model**. Reachability probabilities obtained for the stable states are close to those provided by Avatar. While MaBoSS is clearly faster than Avatar, it is unable to assess complex attractors being thus applicable only when attractors are known to be stable states.

Prism is a model checker that supports probabilistic reachability queries (Kwiatkowska et al., 2011, 2017). To compare Avatar and Prism, we repeated the analysis of the **segment polarity model** with 2 cells. Results are provided in Table 4. Notably, Prism is extremely efficient to evaluate the number of reachable states, a feature not provided by Avatar. Prism performs an exhaustive exploration to evaluate exact reachability probabilities. However, as demonstrated with Avatar, a restricted sample of the dynamics may provide good enough probability estimates in a much shorter time. This feature is particularly useful for larger models. Indeed, for the **sp4 model**, Prism ran out of memory and was thus unable to evaluate the number of reachable states and conclude the analysis (even when increasing the amount of available memory to CUDD to 8Gb).

**Table 4:** Assessing attractor probabilities for the sp2 model with Avatar and Prism.

	Avatar				Prism	
	1E6 runs		1E4 runs			
**Stable states**	**Time**	**Prob.**	**Time**	**Prob.**	**Time**	**Prob.**
SS1		0.8915		0.8921		0.8909
SS2	4h59	0.1084	153s	0.1078	4h25	0.1088
SS3		1.2E-4		1E-4		1.04E-4

*Probabilities returned by* Avatar *are quite similar when considering a lower number of runs, indicating that it is possible to quickly obtain good estimates of reachability probabilities in a much shorter time*.

## 5. Discussion

For models of regulatory networks controlling cell fates, it is of a real interest to identify the model attractors, as well as quantify their reachability over the whole state space or from specific initial conditions. In particular, the impact of model perturbations (e.g., corresponding to observed mutations) on attractors and their basins of attraction has been used to better understand the fates of tumor cells (Huang et al., 2009; Kim et al., 2017; Shah et al., 2018). Most studies rely on Boolean models under a synchronous updating scheme. However, while stable states are identical whatever the updating scheme, it is not the case for the complex attractors, neither for the basins of all attractors. Because the synchronous scheme stems from the assumption that delays associated with component updates are equal, asynchronous updates have been considered more realistic (Thomas, 1991; Abou-Jaoudé et al., 2016). In the context of non-deterministic asynchronous dynamics, it is then relevant to assess the likelihood to reach an attractor and how model perturbations modifies this reachability likelihood. For example, this approach has been used to assess patterns of genetic alterations in bladder tumourigenesis (Remy et al., 2015), or yet to highlight the synergetic roles of Notch gain-of-function and p53 loss-of-function in promoting metastasis (Cohen et al., 2015).

Attractor identification could be achieved by analysing the State Transition Graph (STG) kept in memory but, due to combinatorial explosion, this is impractical for large models. In any case, we are still left with the problem of quantifying attractor reachability in asynchronous dynamics. As an attempt to surpass efficiency bottlenecks and quantification biases of existing methods, we have delineated two novel strategies. Firefront performs a memoryless breath-first exploration of the STG, avoiding any further exploration of states which fall bellow a given threshold α. Avatar performs a modified version of the Monte Carlo algorithm, avoiding the exploration of states previously visited by rewiring and appropriately associating new probabilities with state transitions. To adequately choose the algorithm and optimal values of associated parameters, information about the structure of the dynamics would be needed, which is generally unachievable. Broadly, the breadth of the explored STG and the structure of transient Strongly Connected Components (SCCs) clearly impact Firefront's performances. Avatar's performances are influenced by the degree of connectivity of the SCCs. Ideally, Avatar should avoid to rewire SCCs from which it can easily exit (low connectivity or high exit ratio). On the other hand, it should rewire SCCs from which it is hard to escape. It is also much more efficient to rewire a whole SCC than to iteratively rewire portions of it. While sizes and structures of SCCs are not known *a priori*, Avatar incorporates heuristics that evolve running parameters to the information collected in the course of the simulation.

Results from synthetic and real biological models reveal the ability of Firefront and Avatar to efficiently assess attractor reachability. This type of analysis will permit further biological insights into the dynamics of regulatory and signalling networks. For example, as mentioned above, how model perturbations modify the probability to reach an attractor can reveal the role of single or combined mutations in disease progression. Usage of both algorithms is facilitated through their implementation in GINsim, which provides a convenient graphical user interface.

As future work, the consideration of non-uniform transition probabilities could be easily handled. In particular, when priority classes can be defined by classifying component updates into e.g., slow and fast processes (Fauré et al., 2006), some trajectories are discarded thus modifying the structure of the STG, and therefore the reachability properties. Furthermore, confronting asymptotic model dynamics against experimental time series could provide the ground for model validation.

## Author contributions

CC, PM, JC, and ER designed the research. CC supervised the work, PM supervised the computational implementations, and ER focused on the theoretical foundations. NM specified the algorithms, and implemented the first prototypes. RH revised the algorithms to improve performances, and worked on the GINsim implementation. NM, RH, ER, PM, and CC wrote the paper. All authors reviewed the content of the paper and agreed to endorse it.

### Conflict of interest statement

The authors declare that the research was conducted in the absence of any commercial or financial relationships that could be construed as a potential conflict of interest.

## Footnotes

^1^This process is automated in BoolNetR2GINsim, a small program available at https://github.com/ptgm/BoolNetR2GINsim that accepts user-defined parameters, calls BoolNet and writes the resulting model to a GINML file (the GINsim format).

## Supplementary material

GINsim 3.0 is freely available at http://ginsim.org. The accompanying user documentation, algorithmic details and supplementary results will be made publicly available upon publication.
