# Supplementary material for: Estimating Attractor Reachability in Asynchronous Logical Models
Source: Front Physiol. 2018 Sep 7;9:1161. doi: 10.3389/fphys.2018.01161 (PMC6137237; doi:10.3389/fphys.2018.01161)
Supplement: Supplementary file 1 [file Data_Sheet_1.PDF]

Estimating attractor reachability in asynchronous logical models

SUPPL. MAT. 1: Description of FIREFRONT & AVATAR

N. D. Mendes, R. Henriques, E. Remy, J. Carneiro, P. T. Monteiro, C. Chaouiya

Below we present a more detailed description of the FIREFRONT and AVATAR.

1 FIREFRONT

FIREFRONT is detailed in Algorithm 1. We use the following notation:

- S – the state space
- i – the iteration number
- $i_{\max}$ – the maximum number of iterations
- $N \subseteq S$ – the set of neglected states
- $F \subseteq S$ – the current firefront
- $F' \subseteq S$ – the future firefront
- C – map to count the number of times each firefront was visited
- $\pi[v]$ – the probability associated with $v \in S$
- $v_0 \in S$ – the initial state, from which the algorithm starts the exploration
- A – the set of attractors
- $0 < \alpha < 1$ – the probability threshold to decide whether a state is to be explored
- $\beta \geq \alpha$ – the minimum residual probability allowed in F

At iteration i, the algorithm computes the set of successors of each $v \in F$. If v has no successors, it is moved to A, otherwise, for each successor v', the algorithm verifies whether it belongs to F', N or A. If v' is already present in one of these sets, $\pi[v']$ is updated by adding $\pi[v]$ divided by the number of successors of v, to reflect the contribution of the incoming transition, otherwise, $\pi[v']$ is simply set to $\pi[v]$ divided by the number of successors of v. Based on the value of $\pi[v']$ and the set membership of v', the algorithm then decides whether v' will be part of the future firefront F', or if it is placed/kept in N or A.

After running the algorithm, a set of attractors A (possibly empty) is obtained. For each attractor, $a \in A$, a lower bound of the probability of reaching it from the initial state v is given by $\sum_{v \in a} \pi[v]$. An upper bound is obtained by adding to this value the probabilities of states in F and N after completing the exploration.

FIREFRONT is well-prepared to discover stable states and it is generally capable of discovering multiple complex attractors with dozens to hundred of states. FIREFRONT

Algorithm 1 FIREFRONT exploration algorithm

Input: $\alpha, \beta, v_0, i_{\max}$
Output: A

```
π[v_0] ← 1
F ← {v_0}
N, A ← ∅
i ← 0
C ← ∅
while i < i_max ∧ (∑_{w∈F} π[w]) > β do
    F' ← ∅
    while F ≠ ∅ do
        v ← element of F
        F ← F \ {v}
        if Succ(v) = ∅ then
            A ← A ∪ {v}
        else
            for all v' ∈ Succ(v) do
                if v' ∈ F' ∨ v' ∈ N ∨ v' ∈ A then
                    π[v'] ← π[v'] + π[v]p(v) where by default p(v) = 1/|Succ(v)|
                else
                    π[v'] ← π[v]p(v) where by default p(v) = 1/|Succ(v)|
                end if
                if π[v'] ≥ α then
                    if v' ∉ A then
                        F' ← F' ∪ {v'}
                    end if
                    if v' ∈ N then
                        N ← N \ {v'}
                    end if
                else
                    N ← N ∪ {v'}
                end if
            end for
        end if
    end while
    F ← F'
    if hash(F) ∈ C then
        C(hash(F)) ← C(hash(F)) + 1
    else
        C ← C ∪ {hash(F), 1}
    end if
    if C(hash(F)) > 3 /*firefront exact same content seen at least two times*/ then
        while F ≠ ∅ do
            v ← element of F
            A ← A ∪ complex attractor from successors of v (up to i/3 depth)
            F ← F\a
        end while
    end if
    i ← i + 1
end while
```

might not be able to detect larger or highly intertwined complex attractors for which the oscillating behaviour may not be observed within the pre-specified maximum number of expansions (depth). However, whenever the complex attractors are known, the algorithm is very efficient to calculate the probability of reaching each of them. To this effect, is suffices to provide an oracle which is able to test whether a state belongs to a given known complex attractor. In our implementation, we consider an oracle as a single entity accumulating the probability of all the states recognized by it.

FIREFRONT might miss an attractor whenever the probability to reach this attractor is residual or when the length of the minimum path towards the attractor is higher than the specified maximum number of expansions (depth).

2 AVATAR

The Monte Carlo method is used to estimate the likelihood of outcomes through stochastic simulations. For the problem of attractor quantification, this translates into performing several random walks along the state transition graph, (S, T), from a given initial condition, and taking note of the number of times one reaches each attractor. In its classical formulation, the algorithm does not detect complex attractors and it may not terminate. Therefore, a maximum number of iterations must be specified in order to guarantee the termination of the procedure.

AVATAR described below, consists in modifying the Monte Carlo method to identify the presence of Strongly Connect Components (SCCs) and thus to avoid revisiting a path in the State Transition Graph (STG). Thus, most importantly, AVATAR identifies complex attractors.

Here we provide a detailed description of AVATAR, outlined in Algorithm 2 and associated procedures, using the following notation:

- $v_0 \in S$ – the initial state
- π – a table with transition probabilities, which in the absence of further knowledge it is initially implicitly specified using uniform probabilities such that for any states $v, v' \in S$,
$$\pi(v, v') = \begin{cases} \frac{1}{|\text{Succ}(v)|} & \text{if } (v, v') \in T \\ 0 & \text{otherwise} \end{cases}$$
- Q – the set of states towards which the simulation can progress with non-zero probability, i.e., the successor states
- $u \in S$ – the previously visited state, of which $v \in S$, if any, is a successor
- t – the discovery time for the current incarnation
- C^t – the set of states corresponding to the cycle identified during incarnation t
- D – the set of all visited states
- A – the set of attractors
- transients – the set of all discovered transients
- temporarySCC – transient candidate
- $\delta[v]$ – the discovery time of $v \in S$

Algorithm 2 AVATAR

Input: v_0 /*selected from initial condition*/
τ /*maximal depth for cycle exploration */
A /*previously discovered attractors*/
π /*successor probabilities */
Output: reached attractor

```
t ← 0
F ← {w ∈ S | π(v_0, w) > 0}
Q ← ∅
D ← ∅
temporarySCC ← ∅
transients ← ∅
v ← v_0
while F ≠ ∅ do
    v′ ← random state ∈ F taken with probability π(v, v′)
    if keepTransients then
        for all T ∈ transients do
            if v′ ∈ T then
                v′ ← exit state selected according to exit probabilities
            end if
        end for
    end if
    for all attr ∈ A do
        if v′ ∈ attr then
            return attr
        end if
    end for
    if v′ ∈ D then
        C^t ← {w ∈ D | δ[v′] ≤ δ[w]}
        C^t ← GrowCycle(τ, C^t)
        t ← t + 1
        Q ← {w ∈ S \ C^t | π(w′, w) > 0,    w′ ∈ C^t}
        if Q == ∅ then
            C* ← CalculateComplexAttractor(C^t, temporarySCC)
            return C*
        end if
        RewireGraph(C^t, Q)
        if |C^t| > minTransientSize then
            temporarySCC ← temporarySCC ∪ C^t
        end if
        for all u ∈ C^t do
            δ[u] = t
        end for
        D ← D ∪ C^t
        F ← {w ∈ S | π(v′, w) > 0}
    else
        δ[v′] ← t
        D ← D ∪ {v′}
        F ← {w ∈ S | π(v′, w) > 0}
        if F == ∅ then
            transients ← transients ∪ temporarySCC
            return {v′}
        end if
    end if
    v ← v′
end while
```

If the current state v has never been visited before, the simulation proceeds much in the same way as with the classical MC method. If the state v has no successors and no rewired transitions (*i.e.*, $\forall w \neq v$, $\pi(v,w) = 0$), v is reported as a stable state. If v has been visited before, there is an attempt to extend the discovered cycle with new states. If the enlarged cycle has no exits, the associated complex attractor is inferred and a new iteration is performed. Otherwise, the resulting graph is rewired and the iteration continues with a new incarnation of the STG.

The graph rewiring is presented in Algorithm 3, in which matrix q represents the transition probabilities between states of the cycle (C_t), and r represents the transition probabilities between the states of the cycle and each of the cycle exits (Q), possibly none. r^1 represents the re-computed probabilities for reaching the cycle exits from each of the states in C_t. The theoretical justification for this procedure is presented in the main manuscript (Section 2.3.2).

Algorithm 3 RewireGraph

Input: *Cycle* /*C_t*/, *Exits* /*Q*/
$q \leftarrow [\pi(v,w)]_{v,w \in Cycle}$
$r \leftarrow [\pi(v,w)]_{v \in Cycle, w \in Exits}$
$r^1 \leftarrow \left(\mathrm{Id}_{|Cycle| \times |Cycle|} - q\right)^{-1} r$
for all $v \in Cycle$ **do**
    for all $w \in Cycle$ **do**
        $\pi(v,w) \leftarrow 0$
    end for
    for all $w \in Exits$ **do**
        $\pi(v,w) \leftarrow r^1_{v,w}$
    end for
end for

In order to guarantee an heightened memory efficiency, only a sparse representation of the updated π entries is maintained in memory. In this way, the size of π corresponds to the number of transitions that were rewired at least once. In this context, the probability of transiting from a state u to a state v is either $\pi(u,v)$ if already defined, or the corresponding (uniform) probability if v is a successor of u.

Algorithm 4 GrowCycle

Input: τ, C^t
repeat
    prevCycleSize $\leftarrow |C^t|$
    newStates $\leftarrow \emptyset$
    $Q \leftarrow \emptyset$ /*exits*/
    ExtendCycle(null, C^t, Q, newStates, t, τ, t)
    $\tau \leftarrow \tau * 2$
    $C^t \leftarrow C^t \cup$ newStates
    for $v \in$ newStates **do**
        for $w \in \{s \in S \mid \pi(v,s) > 0\}$ **do**
            if $w \notin C^t$ then $Q \leftarrow \{w\}$
        end for
    end for
    exitRatio $\leftarrow |Q|/|C^t|$
    if $|C^t| <$ maxStatesToRewire **then**
        cycleToRewire $\leftarrow C^t$
    end if
until exitRatio$> 0 \wedge$ prevCycleSize$< |C^t| \wedge |C^t| <$maxStatesToExpand
return cycleToRewire

As described in the main manuscript, an alternative rewiring strategy, Uniform exit probabilities, is available to surpass the memory complexity of the default strategy, Exact exit probabilities. In this alternative rewiring schema, uniform probabilities are associated to the transition from states within the cycle C^t to its exits Q; in this way, there is no need to populate the π transition matrix but simply maintain pairs of the encountered (C^t, Q). When two states u and v satisfy $u \in C^t \wedge v \in Q$ then $\pi(v,w)=\frac{1}{|Q|}$. Although this alternative does not guarantee that the asymptotic probabilities of the

discovered attractors equal their true reachability, it only requires residual time and memory complexity.

In a model generating dynamics with large transients, many intertwined cycles are likely to be encountered in the course of a simulation. To avoid successive costly graph rewirings, an optimization step is introduced. Whenever a cycle is found, a preliminary local exploration procedure is performed prior to the graph rewiring. This procedure is described in Algorithms 4 and 5. Briefly, the neighbouring states of the current cycle are explored up to a maximum depth of τ to verify whether a larger transient cycle can be identified. This procedure is an adaptation of Tarjan's SCC identification algorithm, in which we treat the original cycle as a single state and we limit the exploration by only considering paths of length up to τ.

Algorithm 5 ExtendCycle (adapted version of Tarjan's SCC algorithm)

```
Input: v, Cycle /*C^t*/, Exits /*Q*/, newStates, t, τ, t_0
Q ← Exits
if v = nil then
    for all u ∈ Cycle do
        δ[u]=t
        Q ← Q ∪ {w ∈ S | π(u, w) > 0}
    end for
else
    δ[v]=t
    newStates ← newStates ∪ {v}
    Q ← Q ∪ {w ∈ S | π(v, w) > 0}
end if
t ← t + 1
Additions ← ∅
if τ > 0 then
    for all w ∈ Q do
        if δ[w] is undefined then
            /*w never seen before */
            ExtendCycle(w, Cycle, ∅, newStates, t, τ-1, δ, t_0)
            if v ≠ nil then
                δ[v] = min(δ[v], δ[w])
            end if
        else if v ≠ nil then
            /*w was seen before, and v is not the initial cycle */
            δ[v] = min(δ[v], δ[w])
        end if
    end for
end if
if v ≠ nil∧ δ[v]>t_0 then
    /*if descendants of v never reach the cycle */
    newStates ← newStates \ {v}
end if
```

Since graph rewiring operations may have intervened, we cannot know whether a cycle with no exits corresponds to a complex attractor. To this end, we need to revisit all the cycles that share at least one state with the extended cycle. Algorithm 6 performs this operation by computing the set of states that have ever shared a cycle with u and, recursively, the states that have shared a cycle with them, i.e., the set C^* inductively defined by $u \in C^*$ and $\forall w \in C^* \left(\exists k : w \in C^k \implies C^k \subseteq C^*\right)$. To lessen the incurring computational complexity, Avatar implementation provides an alternative to this step by guaranteeing that a growing cycle incorporates the rewired portions that share at least one state.

Algorithm 6 CalculateComplexAttractor

Input: C^t, ${C^k}_k$
Output: C^*

$C^* \leftarrow \emptyset$
$L \leftarrow C^t$
while $L \neq \emptyset$ **do**
    $u \leftarrow$ a state in L
    $L \leftarrow L \setminus \{u\}$
    $C^* \leftarrow C^* \cup \{u\}$
    $L \leftarrow L \cup \{w \in C^k \setminus \{u\} \mid \exists k : u \in C^k\}$
end while
