# Supplementary material for: Estimating Attractor Reachability in Asynchronous Logical Models
Source: Front Physiol. 2018 Sep 7;9:1161. doi: 10.3389/fphys.2018.01161 (PMC6137237; doi:10.3389/fphys.2018.01161)
Supplement: Supplementary file 2 [file Data_Sheet_2.PDF]

# Estimating attractor reachability in asynchronous logical models

SUPPL. MAT. 2: User documentation of FIREFRONT & AVATAR

N. D. Mendes, R. Henriques, E. Remy, J. Carneiro, P. T. Monteiro, C. Chaouiya

## Download and Installation

### Download

GINsim software (executable GUI and Java source code) is available at http://ginsim.org.

### Requirements

- Java version 8 (JDK 8/JRE 8) or superior. At least 2 GB of free RAM is suggested for the exhaustive analysis of complex models;
- GNUplot 4.2 or superior for plotting facilities.

### Installation Notes

- **GUI**: to run the desktop GUI either double-click directly on the provided .jar file or use the following command on your Linux/OS X/Windows console: `java -jar GINsim-[version].jar`, where [version] should be replaced by the downloaded version. To define the maximum available memory add the parameter -Xmx[memory]m to the previous command where memory defines the number of allocated memory (e.g. `java -jar -Xmx2048m GINsim-3.0.0.jar` to allocate 2GB).
- **API**: to use the programmatic interface within your Java project: 1) add the provided GINsim-[version].jar as a library, or 2) unzip the provided source-code, and directly include the source Java files in your Java project.

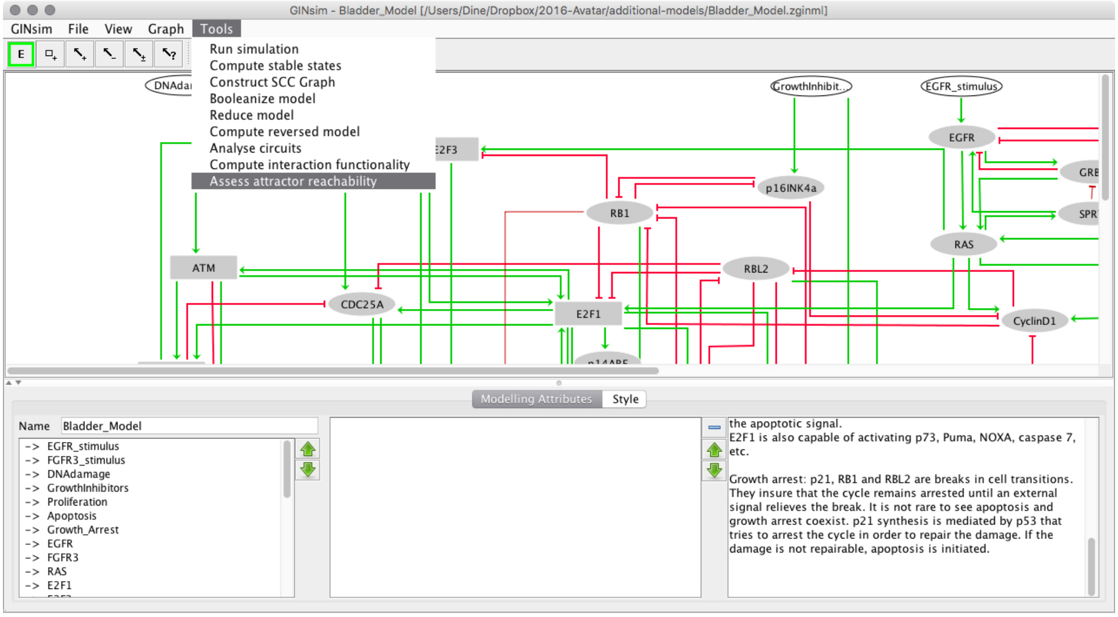


Figure 1: GINsim Tools menu to launch the reachability analysis of the attractors for the current model.

## Running

### GUI

Figure 1 shows the main GINsim window with a model, and how to launch the tool to assess the attractor reachability of that model. Figure 2 shows the window in which the user can select and parametrise Avatar, Firefront and MonteCarlo. The desktop GUI is compatible with Linux/OS X/Windows operating systems, support multiple model formats, and provides several algorithms and visual representations of their outputs.

To run Avatar, Firefront and MonteCarlo algorithms, select in the menu Tools, the menu entry Assess attractor reachability. A new window will appear where the algorithm and associated parameters can be selected (Figure 2). The Run button executes the selected algorithm over the current model and displays the progress of the algorithm at the bottom of the window. The user can force the termination of the algorithm by clicking on the Force exit button.

### API

The API is accompanied with the javadoc documentation and with customizable testing classes to parametrise and run the algorithms. The use of the API is suggested when including the Avatar, Firefront and MonteCarlo algorithms within the context of another tool. This will permit an easy parametrisation, adaptation or extension of the proposed simulations.

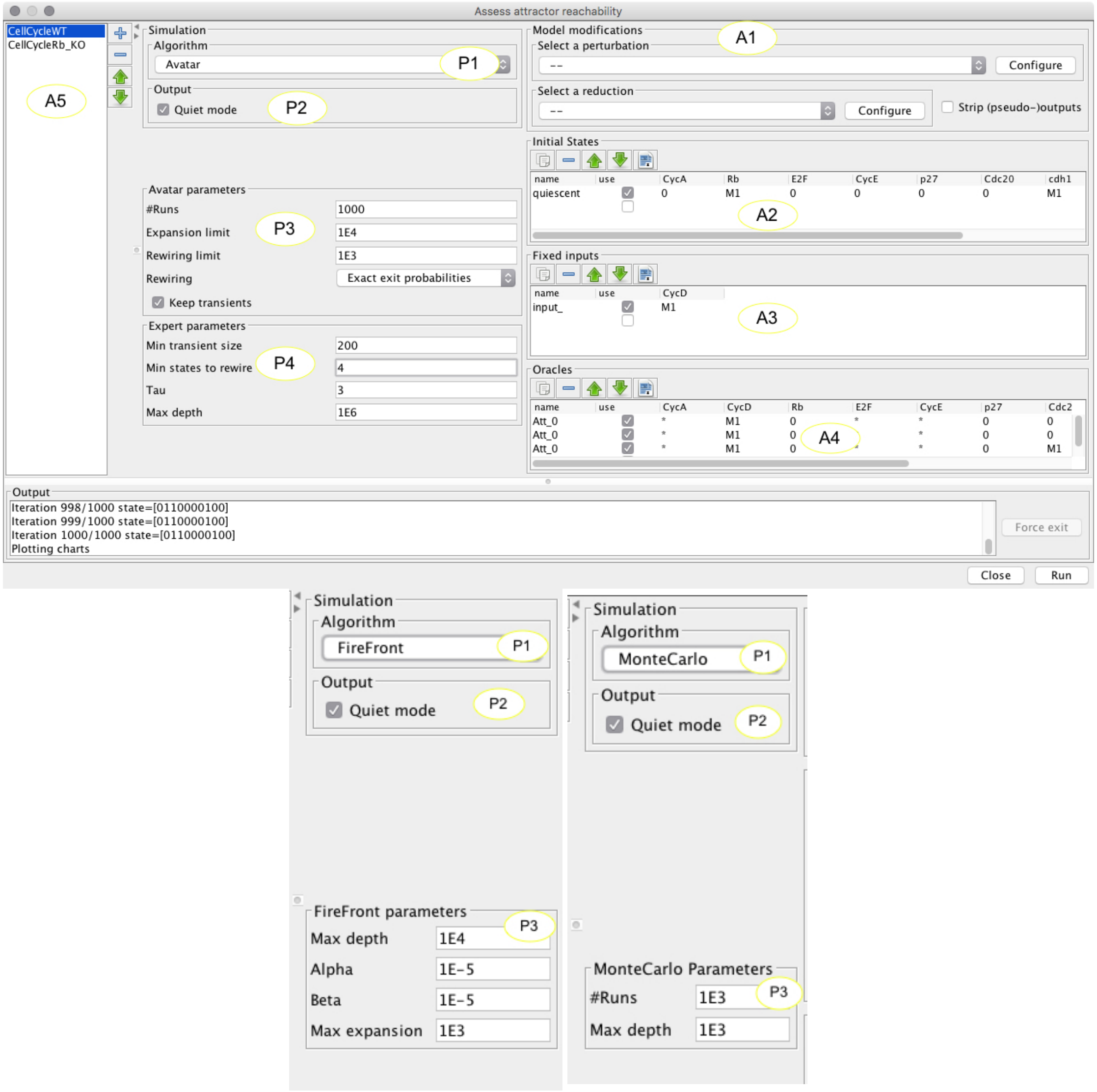


Figure 2: Parameter specification for AVATAR, FIREFRONT and MONTECARLO algorithms.

## Parametrisation

**Model modifiers and initial conditions** The following parameters are recurrent over the main GINsim tools, notably the construction of the State Transition Graph through the simulation tool (see GINsim documentation for details[1]):

- Model perturbations through the definition of knock-outs, ectopic expressions or interaction inactivations ([A1] in Figure 2);
- Model reduction, possibly restricted to the output and pseudo-output components ([A1] in Figure 2);
- Initial states of internal components ([A2] in Figure 2);
- Values of input components ([A3] in Figure 2);

**Oracles** Providing known patterns describing complex attractors can greatly improve the efficiency of AVATAR and FIREFRONT ([A4] in Figure 2).

[1] `http://ginsim.org/documentation`

**Algorithm** Choice of AVATAR, FIREFRONT or MONTECARLO algorithms to identify the attractors and assess their reachability probabilities ([P1] in Figure 2). AVATAR should be preferred for models with large transient SCCs and complex attractors, whereas FIREFRONT may provide more precise evaluations for remaining models. MONTECARLO can be a good option for a fast assessment, provided the dynamics does not include complex attractors.

**Output** Choice whether detailed logs are to be printed or not. Note that selecting the non-quiet mode hampers efficiency (suggested for debugging purposes only) ([P2] in Figure 2).

AVATAR **parameters**
A first set of parameters include ([P3] in Figure 2):

- **#Runs**: number of iterations;
- **Expansion limit**: minimum number of states in a SCC to stop expansion;
- **Rewiring limit**: maximum number of states in a SCC to be rewired in a single step;
- **Rewiring strategy**: Exact exit probabilities performing random matrix inversions in the course of cycle rewiring (prone to memory bottlenecks) *versus* Uniform exit probabilities performing the same rewiring but associating uniform probabilities to cycle exits (efficient, yet reachability quantification may be biased);
- **Keep Transients**: whether transient cycles should be kept between iterations – suggested `true`, in particular when choosing Exact exit probabilities strategy;

The second set of parameters may be subtler to handle ([P4] in Figure 2):

- **Min transient size**: minimum size of transient SCCs kept in memory for subsequent runs;
- **Min states to rewire**: minimum required number of states withing a cycle for rewiring;
- **Tau**: cycle expansion rate: $\tau = 3 \Rightarrow 6 \Rightarrow 12 \Rightarrow 24 \ldots$
- **Max Depth**: maximum number of visited states per simulation.

FIREFRONT **parameters**
Parameters include ([P3] in Figure 2):

- **Max depth**: limit depth (number of expansions), prevents a simulation to get locked within large SCCs;
- **Alpha**: the minimum probability for a state to be expanded; states with probability below $\alpha$ are moved to the neglected set $N$;
- **Beta**: the maximal residual probability of FIREFRONT set $F$ to stop simulation;
- **Max expansion**: maximum number of states to expand per iteration (non-expanded states are preserved for upcoming expansions).

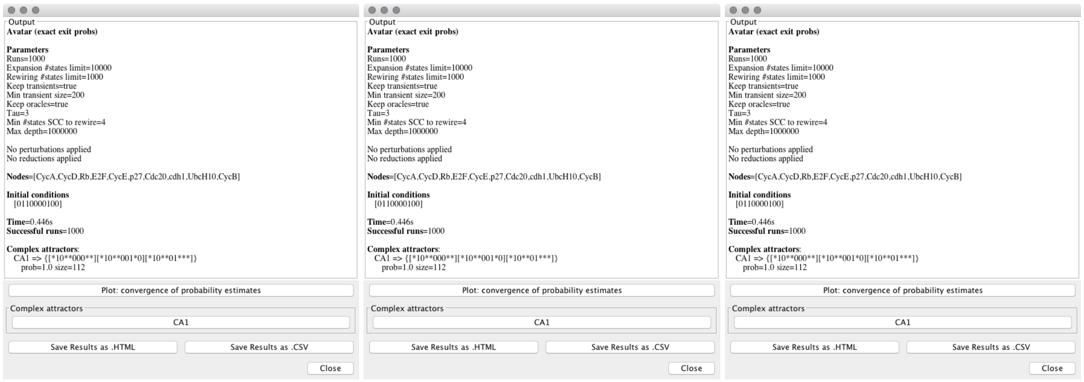


Figure 3: Textual outputs of AVATAR (left), FIREFRONT (middle) and MONTECARLO (right).

### MONTECARLO parameters

Parameters include ([P3] in Figure 2):

- **#Runs**: number of iterations;
- **Max depth**: limit of the depth (*i.e.*, number of visited states) for each simulation; prevents a simulation to get locked in a terminal or transient SCC.

**Saving parametrisation** Parameters for assessing attractor reachability can be defined (through the parameter definitions), removed, selected and modified using the left panel of the GUI ([A4] in Figure 2). These are saved along the model (*i.e.*, are stored in the GINsim .zginml archive).

## Output

### Textual Display

The results of the algorithms are displayed, describing the attractors found, their reachability probabilities as well as additional information dependent on the selected algorithm (see Figure 3).

### Plots

In addition to the textual results, AVATAR and FIREFRONT provide visualisation of specific information as plots:

- AVATAR plots the progression of the probability estimates per attractor along the iterations;
- FIREFRONT plots the evolution of the cardinals and of the cumulative probabilities of the sets $F$, $N$ and $A$.

**Note**: GINsim plotting facilities rely on the JavaPlot library[2], which requires GNUplot[3] or superior to be previously installed.

[2] http://javaplot.panayotis.com

[3] https://sourceforge.net/projects/gnuplot/
