# Supplementary material for: Estimating Attractor Reachability in Asynchronous Logical Models
Source: Front Physiol. 2018 Sep 7;9:1161. doi: 10.3389/fphys.2018.01161 (PMC6137237; doi:10.3389/fphys.2018.01161)
Supplement: Supplementary file 3 [file Data_Sheet_3.PDF]

Estimating attractor reachability in asynchronous logical models

SUPPL. MAT 3: Dynamics structure for random2, synthetic1 and synthetic2 models

N. D. Mendes, R. Henriques, E. Remy, J. Carneiro, P. T. Monteiro, C. Chaouiya

Compact representations of model dynamics are provided here in the form of Hierarchical Transition Graphs (HTG) to support results of main text Table 2 for the models random1, synthetic1 and synthetic2. Going beyond the well-known SCC condensation, HTGs correspond to new condensations, which better reveal the organisation of STGs in terms of basins of attraction [1]. Briefly, the nodes in HTG gather states which are part of the same SCC or which are irreversible (that cannot be visited twice) and share the same reachable properties of dowstream SCCs and attractors.

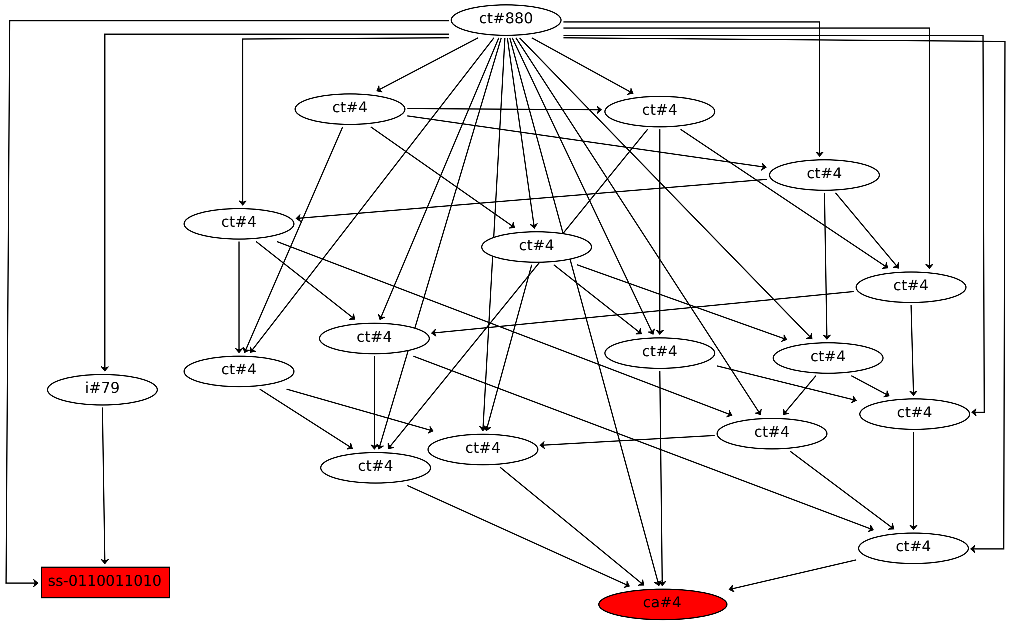

Figure 1: Hierarchical Transition Graph of the model random1: 1024 states are reachable from the initial state, which is part of a Strongly Connected Component (SCC) of 880 states (top node labelled ct#880). The remaining of the dynamics encompasses 1) 15 SCCs with 4 states each (nodes ct#4) towards the cyclic attractor (ca#4), which includes 4 states; 2) linear trajectories made of 79 irreversible states gathered into node i#79.

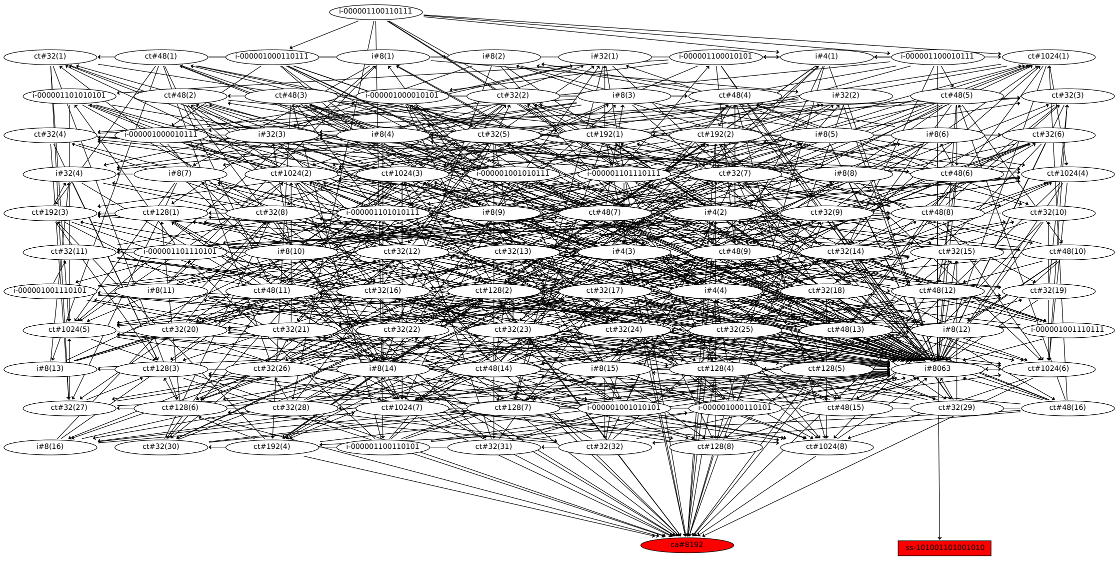

Figure 2: Hierarchical Transition Graph of the model synthetic1: 28320 states are reachable from the initial state. The dynamics encompasses 68 transient SCCs of variable sizes (from 32 to 1024 states), ending in a stable state or a large complex attractor of 8192 states.

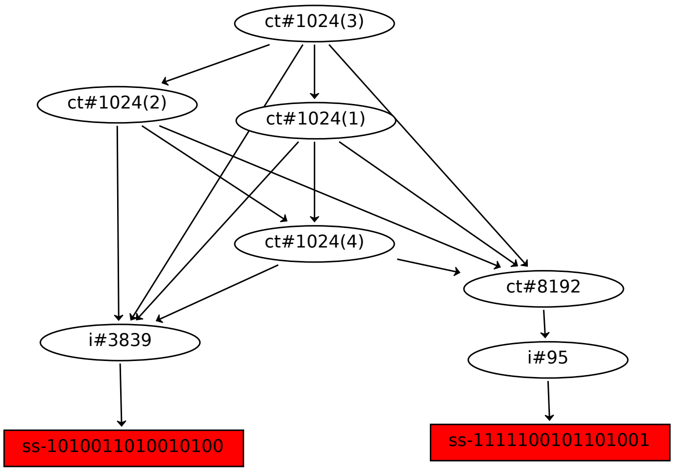

Figure 3: Hierarchical Transition Graph of the model synthetic2: 16224 states are reachable from the initial state. The dynamics encompasses 5 transient SCCs; 4 of them contain 1024 states and are in the common basin of attraction of the 2 stable states. From there, trajectories reaching the stable state on the left go through a large SCC of 8192 states, whereas trajectories leading to the stable state on the right go through linear paths of irreversible states.

Reference

[1] Bérenguier D, et al. Dynamical modeling and analysis of large cellular regulatory networks. Chaos, 23(2):025114, 2013
