# Supplementary material for: Estimating Attractor Reachability in Asynchronous Logical Models
Source: Front Physiol. 2018 Sep 7;9:1161. doi: 10.3389/fphys.2018.01161 (PMC6137237; doi:10.3389/fphys.2018.01161)
Supplement: Supplementary file 4 [file Data_Sheet_4.PDF]

Estimating attractor reachability in asynchronous logical models

SUPPL. MAT. 4: Impact of α's values on FIREFRONT results illustrated

N. D. Mendes, R. Henriques, E. Remy, J. Carneiro, P. T. Monteiro, C. Chaouiya

Here we consider a logical model of the regulatory network and signalling pathways controlling Th cell differentiation [1]. The model stable states correspond to canonical Th1, Th2, Th17 and Treg subtypes, in addition to other transient hybrid cell types that co-express combinations of Th1, Th2, Treg and Th17 markers. This model encompasses 78 components, leading to a space of over 10^{20} states. It is available in the Supplementary Archive containing the models used in this work (`th_cell.zginml`). Here, we performed a reachability analysis with FIREFRONT to assess the stable states reachable from a naive cell (Th0) under a pro-Th2 environment (see [1]). The 3 reachable stable states correspond to Th2, and the tables show the values of the components as in Figure 6 in [1]. Figure 1 illustrates how FIREFRONT's parameters influence the quality of the results.

FIREFRONT, 1st set of parameters

Alpha 1E-05
Beta 0.0001
Max depth 1000000
Max states expanded/Iteration 1000

Time 31.567 secs

SS1	IL2R	IL2RA	IFNG	IL2	IL4	IL10	IL21	IL23	TGFB	TBET	GATA3	FOXP3	NFAT	STAT1	STAT3	STAT4	STAT5	STAT6	proliferation	RORGT	IL17	Probability lowerbound	Probability upperbound
SS2	0	1	0	0	0	0	0	0	0	0	1	0	1	0	1	0	1	1	0	0	0	0.13665	0.94552
SS3	0	1	0	0	1	1	1	1	0	0	1	0	1	0	1	0	1	1	1	0	0	0.00898	0.81786
	0	1	0	0	1	1	1	1	0	0	1	0	1	0	1	0	2	1	1	0	0	0.0455	0.85437

Probability of state sets:
Neglected set: 0.8089
Attractor set: 0.1911

FIREFRONT, 2nd set of parameters

Alpha 1E-06
Beta 0.0001
Max depth 100000
Max states expanded/Iteration 10000

Time 158.211 secs

	IL2R	IL2RA	IFNG	IL2	IL4	IL10	IL21	IL23	TGFB	TBET	GATA3	FOXP3	NFAT	STAT1	STAT3	STAT4	STAT5	STAT6	proliferation	RORGT	IL17	Probability lowerbound	Probability upperbound
SS1	0	1	0	0	0	0	0	0	0	0	1	0	1	0	1	0	1	1	0	0	0	0.52851	0.77152
SS2	0	1	0	0	1	1	1	1	0	0	1	0	1	0	1	0	1	1	1	0	0	0.04333	0.28634
SS3	0	1	0	0	1	1	1	1	0	0	1	0	1	0	1	0	2	1	1	0	0	0.18515	0.42816

Probability of state sets:
Neglected set: 0.243
Attractor set: 0.757

Figure 1: By considering a lower value of α and higher number of expansion, FIREFRONT provides better results (with a higher total probability in the attractor set). However, the neglected set still gathers a quarter of the total probability, which implies large ranges for the attractors' probabilities. Lowering further α may lead to better results, but because of the model size, AVATAR is more adequate (see Figure 2).

AVATAR

Runs 100000
Expansion #states limit 10000
Rewiring #states limit 1000
Keep transients true
Min transient size 200
Keep oracles true
Tau 3
Min #states SCC to rewire 4
Max depth 100000

Time 1125.668 secs

	IL2R	IL2RA	IFNG	IL2	IL4	IL10	IL21	IL23	TGFB	TBET	GATA3	FOXP3	NFAT	STAT1	STAT3	STAT4	STAT5	STAT6	proliferation	RORGT	IL17	Probability
SS1	0	1	0	0	0	0	0	0	0	0	1	0	1	0	1	0	1	1	0	0	0	0.70521
SS2	0	1	0	0	1	1	1	1	0	0	1	0	1	0	1	0	1	1	1	0	0	0.05708
SS3	0	1	0	0	1	1	1	1	0	0	1	0	1	0	1	0	2	1	1	0	0	0.23771

Max transient size 6
Successful runs 100000

Figure 2: Probabilities obtained by AVATAR.

Reference

[1] Naldi A, et al. Diversity and plasticity of Th cell types predicted from regulatory network modelling. PLoS Comput Biol, 6(9):e1000912, 2010.
