# Supplementary material for: Estimating Attractor Reachability in Asynchronous Logical Models
Source: Front Physiol. 2018 Sep 7;9:1161. doi: 10.3389/fphys.2018.01161 (PMC6137237; doi:10.3389/fphys.2018.01161)
Supplement: Supplementary file 5 [file Data_Sheet_5.PDF]

Estimating attractor reachability in asynchronous logical models

SUPPL. MAT. 5: Impact of mutations on attractor reachability illustrated

N. D. Mendes, R. Henriques, E. Remy, J. Carneiro, P. T. Monteiro, C. Chaouiya

Here we consider a logical model of cellular response upon death receptor engagement to illustrate the biological significance of assessing probabilities of attractors under model perturbations [1]. The model file `cell_fate.zginml` is available in the Supplementary Archive containing the models used in this work. These results are to be compared with Figure 2 of the original publication [1]. Important to note that here, we performed the analysis with AVATAR using the full model, whereas Calzone et al. had to consider a reduced version of their model. This analysis illustrates that model reduction may alter the reachability probabilities of the attractors.

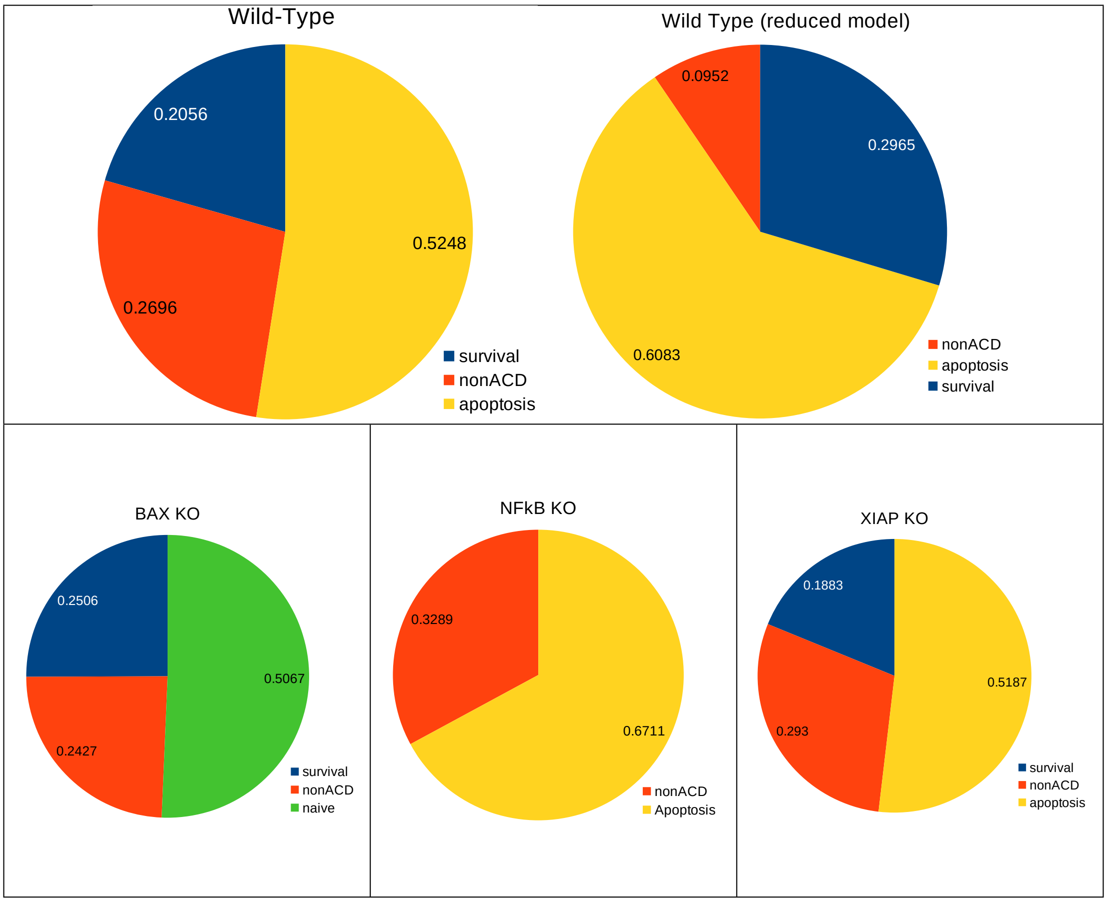

Reference

[1] L. Calzone, L. Tournier, S. Fourquet, D. Thieffry, B. Zhivotovsky, E. Barillot, and A. Zinovyev. Mathematical modelling of cell-fate decision in response to death receptor engagement. PLoS Computional Biology, 6(3):e1000702.
